# AI-powered immune profiling from histopathology slides for chemo-radiotherapy outcome prediction in rectal cancer: a study using clinical trial and real-world cohorts

**DOI:** 10.1016/j.ebiom.2025.105993

**Published:** 2025-11-17

**Authors:** Zhuoyan Shen, Douglas Brand, Mikaël Simard, Adam P. Levine, Sumeet Hindocha, Talisa Mistry, Dahmane Oukrif, Andre Lopes, Rubina Begum, Nicholas P. West, Ying Zhang, Gary Royle, Tim S. Maughan, David Sebag-Montefiore, Maria A. Hawkins, Charles-Antoine Collins-Fekete

**Affiliations:** a Department of Medical Physics and Biomedical Engineering, University College London, London, UK; b Department of Radiotherapy, University College London Hospitals NHS Foundation Trust, London, UK; c Research Department of Pathology, University College London, London, UK; d Department of Cellular Pathology, University College London Hospitals NHS Foundation Trust, London, UK; e Cancer Institute, University College London, London, UK; f Division of Pathology and Data Analytics, Leeds Institute of Medical Research, School of Medicine, University of Leeds, Leeds, UK; g Division of Oncology, Leeds Institute of Medical Research, School of Medicine, University of Leeds, Leeds, UK; h Department of Oncology, University of Oxford, Oxford, UK

**Keywords:** Colorectal cancer, Tumour immune microenvironment, Chemo-radiotherapy, Artificial intelligence, Digital pathology

## Abstract

**Background:**

The impact of the tumour-immune microenvironment on locally advanced rectal cancer (LARC) outcomes remains unclear. This study quantitatively assesses the synergistic influence of tumour-infiltrating lymphocytes (TILs), tumour-associated macrophages (TAMs), mitotic activity, and DNA mutations in predicting outcomes for LARC patients undergoing neoadjuvant chemo-radiotherapy (nCRT).

**Methods:**

Three cohorts (ARISTOTLE-RC, UCLH-RC, TCGA-CRC) were stratified by densities of AI-quantified TILs, TAMs, and mitotic figures with cut-offs identified on a hold-out subset and integrated with DNA mutations to assess correlations with disease-free survival (DFS) and overall survival (OS). Immune cell dynamics pre- and post-CRT were also evaluated.

**Findings:**

In ARISTOTLE-RC, TIL^+^ patients had significantly improved DFS (HR = 0.59, 95% CI: 0.39–0.90, p = 0.013) and OS (HR = 0.42, 95% CI: 0.24–0.73, p < 0.005), while TAM^+^ was associated with shorter DFS (HR = 1.65, 95% CI: 1.00–2.72, p = 0.045). Similar patterns were observed in UCLH-RC and TCGA-CRC. TIL^+^/*KRAS*^−^ patients had significantly improved DFS (HR = 0.41, 95% CI: 0.22–0.75, p < 0.005) and OS (HR = 0.28, 95% CI: 0.13–0.62, p < 0.005). In *TP53*-mutated patients, TAM^+^ group showed shorter DFS (HR = 1.46, 95% CI: 1.07–2.01, p = 0.0151), while among *TP53*-wild-type patients, no difference in DFS (HR = 1.00, 95% CI: 0.66–1.52, p = 0.9930) was observed between the two subgroups. Patients who transitioned after nCRT from TIL^−^ to TIL^+^ had improved DFS (HR = 0.70, 95% CI: 0.50–0.97, p = 0.028) and exhibited a significantly higher pre-treatment mitotic index (mean difference = 9.36, 95% CI: 1.87–16.85, p = 0.0385).

**Interpretation:**

These findings suggest the potential utility of AI-driven immune profiling for clinical decision-making in LARC patients undergoing nCRT.

**Funding:**

10.13039/501100000289Cancer Research UK (RRNPSF-Jan21/100001, A18745, C7893/A2899), 10.13039/100014013UK Research and Innovation (MR/T040785/1).

**Research in context**

**Evidence before this study**

We conducted a systematic review of the PubMed database to identify studies examining the prognostic significance of tumour-infiltrating lymphocytes (TILs), tumour-associated macrophages (TAMs), and mitotic activity in colorectal cancer (CRC), covering the period from May 2015 to May 2025. The search included all languages and used the terms “colorectal cancer” AND “tumour infiltrating lymphocytes”, “colorectal cancer” AND “tumour-associated macrophages”, and “colorectal cancer” AND “mitotic activity”. This yielded 1186, 557, and 133 articles, respectively. Evidence shows that higher TIL density is associated with improved outcomes and may predict response to neoadjuvant chemo-radiotherapy (nCRT). In contrast, findings on TAMs were inconsistent across studies. However, most of the studies were conducted with a limited number of patients and the impact of *KRAS* mutation and nCRT was barely illustrated, nor the pattern dynamics after the nCRT. Studies on mitotic activity were scarce, and rarely used quantitative methods. Additionally, few studies employed artificial intelligence (AI) for automated cell quantification in histopathology, highlighting a gap in scalable, reproducible biomarker analysis.

**Added value of this study**

This study provides evidence that high TIL and low TAM levels are favourable prognosticators for all-stage colorectal cancer patients and locally advanced rectal cancer (LARC) patients who received nCRT in both a clinical trial and real-world clinical settings. We identified TIL density and *KRAS* mutation status as independent predictors of survival, and their combined stratification revealed strong prognostic significance. Furthermore, dynamic changes in TIL density following nCRT were associated with better outcomes in immunoreactive patients. We also found that extremely high mitotic activity is linked to poorer survival and may contribute to immune suppression after nCRT. Notably, this study developed an AI-based framework to automatically quantify these markers from routine histopathology slides, offering a rapid and cost-effective approach for biomarker integration in clinical workflows.

**Implications of all the available evidence**

Our findings highlight the potential for AI-assisted quantification of key components in the tumour immune microenvironment to enhance risk stratification in CRC, especially for LARC patients undergoing nCRT. The automated analysis offers a scalable and reliable method for evaluating TIL and TAM density, as well as mitotic index, in clinical workflows, potentially leading to more personalised treatment strategies and improved patient outcomes. Future research should focus on validating these AI-driven markers in larger, diverse cohorts and exploring their integration with other genomic and molecular markers to identify patients whose immune activity can be triggered by nCRT, and further refine patient selection and treatment planning.

## Introduction

Colorectal cancer (CRC) is the third most common cancer and the second leading cause of cancer-related mortality worldwide.^1^ The charity Bowel Cancer UK highlighted the importance of integrating genomics, big data science and digital pathology methods to improve the morpho-molecular taxonomy and biological stratification of CRC.^2^ Developing biomarkers is crucial to define the optimal curative therapeutic strategy for an individual or group, improving treatment selection and minimising the risk of overtreatment.^3^

Chemo-radiotherapy (CRT) is a common treatment option for rectal cancers, especially locally advanced rectal cancer (LARC).^4^^,^^5^ LARC patients have a higher risk of recurrence, necessitating more intensive neoadjuvant treatment strategies than early-stage rectal cancer patients.^6^ Optimising treatments in LARC involves intensifying treatment (e.g. total neoadjuvant therapy (TNT)) prior to total mesorectal excision (TME).^7^ Trials investigating changes in TNT regimes have been published with evidence that impacts clinical practice.^8^, ^9^, ^10^ Although the management of LARC is advancing, the focus has been largely on modifying the sequencing of treatments rather than the selection of treatments themselves. Combining emerging techniques, including artificial intelligence (AI), digital pathology, and whole genome sequencing (WGS) offers the opportunity to develop advanced signatures for precise treatment selection. Recently, a phase II study reported that LARC patients with mismatch repair deficiency are sensitive to treatment with neoadjuvant PD-1 blockade and can avoid standard CRT and surgery.^11^

The tumour immune microenvironment (TIME) encompasses the whole cellular and acellular environment where tumour cells exist. TIME has a pivotal role in shaping tumour phenotype, evolutionary dynamics, and therapy responses, and its impact on prognosis and treatment response is well-established across human cancers.^12^ Tumour-infiltrating lymphocytes (TILs) and tumour-associated macrophages (TAMs) serve as key indicators of immune activity within the TIME. High TIL is associated with favourable outcomes across a variety of cancer types.^13^, ^14^, ^15^ High TIL density is also a prognostic factor for better treatment response and longer disease-free survival (DFS) after neoadjuvant chemo-radiotherapy (nCRT) for rectal cancer.^16^^,^^17^

The prognostic significance of TAMs remains less clear. Their impact is influenced by phenotypic diversity, as M1-like TAMs are linked to better prognosis due to their pro-inflammatory and tumour-suppressive functions, whereas M2-like TAMs promote tumour progression by enhancing angiogenesis, suppressing immune responses, and facilitating metastasis.^18^ Studies have reported conflicting findings regarding TAMs in CRC. While some studies suggest that high TAM infiltration is linked to improved prognosis in colon cancer,^19^, ^20^, ^21^ others have reported contradictory results.^22^, ^23^, ^24^ Additionally, it has been linked to poorer outcomes in rectal cancer.^25^^,^^26^ This discrepancy highlights the differences in molecular phenotypes and treatment regimens. Notably, the common use of CRT in rectal cancer may alter the TIME and influence TAM behaviour.^27^^,^^28^ However, large-scale studies investigating TAMs in CRT-treated rectal cancer cohorts remain limited.

Tumour proliferation is a critical factor influencing cancer outcomes. The mitotic index (MI), which quantifies mitotic activity within a tumour, is a well-established grading criterion for multiple solid tumours.^29^, ^30^, ^31^ However, limited studies have reported the negative impact of MI or cell proliferation markers on survival in CRC.^32^^,^^33^ In addition, while some studies suggest an association between mitosis and immune activity, the evidence is inconclusive.^34^

In this study, we aimed to characterise the prognostic utility of AI-derived TIL and TAM density through a phase III trial cohort and a real-world clinical cohort undergoing CRT. Immune cell metrics and MI were quantified using an AI framework applied to digitised whole slide images (WSIs) of pre-treatment biopsies and post-treatment resections. Cut-off values were determined within a hold-out sub-cohort, and DFS and overall survival (OS) were compared among stratified patients to evaluate the impact of the immune cell landscape, CRT, tumour proliferation, and DNA mutations.

## Methods

### Study population

Three cohorts were included in this study:

1. ARISTOTLE-RC: 589 participants with MRI-defined LARC from the ARISTOTLE trial (ISRCTN09351447). They were randomised into two treatment arms: A) Standard arm (SCRT)-capecitabine 900 mg/m^2^ orally twice daily Monday to Friday for five weeks with radiotherapy 45Gy in 25 fractions; B) Experimental arm (IrCRT)-irinotecan 60 mg/m^2^ intravenous once weekly for four weeks and capecitabine 650 mg/m^2^ orally twice daily Monday to Friday for five weeks with radiotherapy 45Gy in 25 fractions. 414 patients with available digitised whole slide images (WSIs) were included and divided into sub-cohorts in this study. A diagram of the inclusion and exclusion criteria is shown in Figure S1. Diagnostic years range from 2011 to 2018. Sex data were collected from clinical records as documented by the treating clinical teams.
2. UCLH-RC: A retrospective cohort of 70 MRI-defined LARC patients who received standard CRT at University College London Hospitals NHS Foundation Trust. Diagnostic years range from 2013 to 2022. Sex data were collected from clinical records as documented by the treating clinical teams.
3. TCGA-CRC: A publicly available cohort of 458 colorectal cancer (CRC) patients across stages I–IV from The Cancer Genome Atlas (TCGA).^35^ Diagnostic years range from 1998 to 2013.

Information on the clinical data and digitised WSIs used in each cohort is listed in Table S1. DFS and OS were used as the outcome measures. The follow-up time is up to five years.

### Ethics

Ethical approval for the ARISTOTLE trial was granted by the NHS Health Research Authority (HRA) (REC reference: 10/H0706/65, IRAS ID: 5406). Ethical approval for the UCLH-RC cohort was provided by the UCL/UCLH Biobank for Studying Health and Disease (study EC30.22) under NHS HRA delegated authority (REC reference: 20/YH/0088, IRAS ID: 272816). Written informed consent was obtained from all participants.

### Study design

**Fig. 1:**
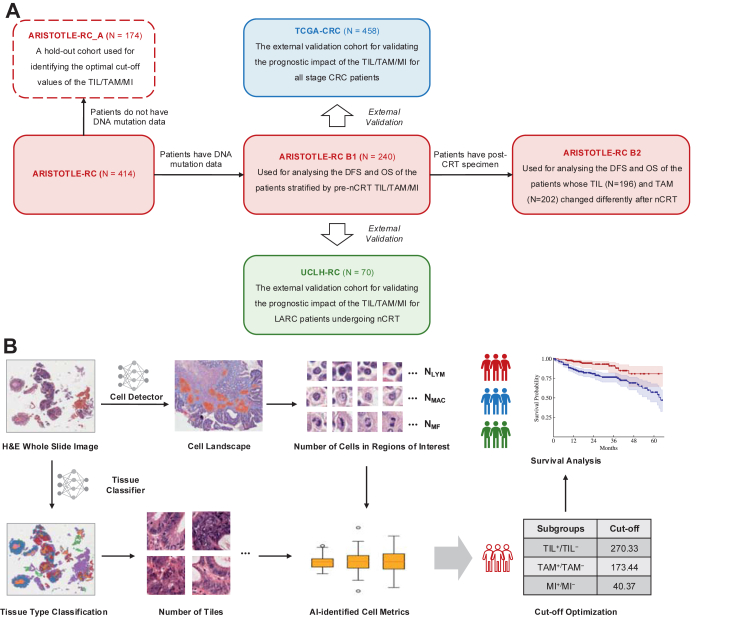
**Illustration of the study design.** A. Description of the cohorts used in this study. B. Workflow of stratifying patients using cell metrics identified by the AI framework.

The study design workflow is illustrated in Fig. 1A. This study is based on a post hoc analysis of the ARISTOTLE trial and was not conducted according to a pre-specified protocol. Within ARISTOTLE-RC, 174 patients without available DNA mutation data were designated as a hold-out cohort (ARISTOTLE-RC A) to determine optimal cut-off values for TIL and TAM density and MI. The optimal values maximise Youden's index^36^ in the receiver operating characteristic (ROC)^37^ analysis targeting the three-year disease progression. ARISTOTLE-RC B1 and ARISTOTLE-RC B2 are used as the validation sets to compare the DFS and OS of the patients stratified by the pre-treatment metrics (B1) and the immune dynamics before and after the treatment (B2). The pre-treatment timepoint was defined as the baseline diagnostic biopsy before initiation of nCRT, and the post-treatment timepoint as the surgical resection specimen. Only patients with matched pre- and post-treatment samples and remaining tumour tissue post-treatment were included in ARISTOTLE-RC B2. *KRAS* and *TP53* mutation status were selected for downstream patient stratification for the high mutation rates. Findings from ARISTOTLE-RC were externally validated using the UCLH-RC cohort. In addition, the TCGA-CRC cohort was used to explore the prognostic value of cell-based metrics across all CRC stages.

### AI framework

An AI framework (Fig. 1B) consisting of a tissue classifier and a cell detector was developed and deployed to automatically detect lymphocytes, macrophages, and mitotic figures in the three cohorts.

The framework consists of two AI models:

(1) Tissue Classifier. An EfficientNetB0^38^ was trained on the NCT-CRC-HE-100 K dataset,^39^ consisting of 100,000 non-overlapping tiles of nine types of tissue: adipose (ADI), background (BACK), debris (DEB), lymphocytes (LYM), mucus (MUC), smooth muscle (MUS), normal colon mucosa (NORM), cancer-associated stroma (STR), colorectal adenocarcinoma epithelium (TUM). The size of a single tile is 224 pixels × 224 pixels with a pixel size of 0.5 μm/pixel. The tissues were manually extracted and labelled from pathologist-annotated regions in the WSIs. The EfficientNet was trained using a single NVIDIA GeForce RTX 3090 GPU for 100 epochs. The training batch size was 256, and the optimiser was AdamW algorithm with a learning rate of 0.0001. Random horizontal flips (p = 0.5) were also used for spatial augmentation.
(2) Cell Detector. A YOLOv10x model^40^ was trained for detecting individual cells of interest. For detecting the immune cells, the model was trained on the Immunocto dataset^41^ with 2,282,818 immune cells labelled by multiplex immunofluorescence. For detecting the mitotic figures, the model was trained on the OMG-Octo^42^ dataset with 74,620 pan-cancer mitotic figures. The yolov 10 model was trained using 4 NVIDIA GeForce RTX 3090 GPUs for 200 epochs. The training batch size was 48, and the optimiser was AdamW algorithm with a learning rate of 0.01. Spatial augmentation was applied, and the configuration parameters are listed in Table S4.

Colour augmentation was applied to the training data for both models to reduce the impact of the staining variation and increase the robustness of the model. RGB images were first deconvolved into haematoxylin (H) and eosin (E) channels using the stain separation method proposed by Ruifrok and Johnston.^36^ To introduce realistic stain variation, we employed the stain concentration perturbation scheme described by Tellez et al.,^43^ where perturbations were uniformly sampled (σ = 0.14) and applied to the deconvolved H&E channels before reconstructing the RGB images. The data size for training, validation and testing, model performance on detecting tumours and different types of cells are listed in Tables S3 and S5. Examples of outputs from the framework are illustrated in Figure S2.

The WSIs are first analysed by the tissue classifier to identify regions of interest (ROIs) for analysing TILs, TAMs, and MI. Lymphocytes and mitotic figures are then detected by the cell detector within tumour regions, while macrophages are detected in both tumour and stromal regions. AI model development and digital pathology data analyses were conducted blinded to clinical outcomes.

### Definition of the measurements and notations

In clinical practice, no robust method has been established for directly quantifying TILs and TAMs in H&E-stained slides. In this study, we reported cell densities per 10 High-Power-Field (2 mm^2^) for evaluating the TIL, TAM, and MI within a WSI, to maintain consistency with our previous work on AI-assisted mitotic index assessment.^42^

The metrics were defined by Equations Equation 1, Equation 2, Equation 3, where $N_{lym}$, $N_{mac}$ and $N_{mf}$ are the number of detected lymphocytes, macrophages and mitotic figures, respectively. $N_{TUM}$ and $N_{STR}$ are the number of tumour tiles and the number of stroma tiles, $A_{10HPF}$ is the area of 2 mm^2^ and *A_tile_* is the area of a single tile (1.25 × 10^−2^ mm^2^).

Equation 1 $$TIL=\frac{N_{lym}}{N_{TUM}\;\times \;A_{tile}}\;\times \;A_{10HPF}$$

Equation 2 $$TAM=\frac{N_{mac}}{\left(N_{TUM}\;+\;N_{STR}\right)\;\times \;A_{tile}}\;\times \;A_{10HPF}$$

Equation 3 $$MI=\frac{N_{mf}}{N_{TUM}\;\times \;A_{tile}}\;\times \;A_{10HPF}$$

Table 1 lists the cut-off values for stratifying patients by the density metrics and the notations used to represent different subgroups in this paper. The optimisation of the cut-off values was done by ten-fold cross-validation using the data of ARISTOTLE-RC A.

**Table 1:** Notations representing patients stratified by cell density.

Notation	Subgroup	Cut-off (cells per 2 mm^2^)
TIL^+^	Patients with high pre-treatment TIL density	270.33
TIL^−^	Patients with low pre-treatment TIL density	
TAM^+^	Patients with high pre-treatment TAM density	173.44
TAM^−^	Patients with low pre-treatment TAM density	
MI^+^	Patients with high pre-treatment mitotic figure density	40.37
MI^−^	Patients with high pre-treatment mitotic figure density	
TIL/TAM^++^	Patients whose TIL/TAM density remained high after the treatment	–
TIL/TAM^−+^	Patients whose TIL/TAM density remained low after the treatment	–
TIL/TAM^+−^	Patients whose TIL/TAM density changed from high to low after the treatment	–
TIL/TAM^−−^	Patients whose TIL/TAM density changed from low to high after the treatment	–

### Statistics

Survival analysis was conducted using Python (v3.9.13) and the lifelines library (v0.28.0). Kaplan-Meier^44^ estimation was performed to generate survival curves, and differences between subgroups were assessed using the log-rank test.^45^ Hazard Ratios (HR) with a 95% confidence interval (CI) were given by the Cox proportional hazard model.^46^ Pearson test, Chi-square test,^47^ Mann–Whitney test^48^ and Kruskal–Wallis test^49^ were performed using SciPy library (version = 1.13.1) for correlation and differentiation analysis. All p-values are two-sided, and a p-value of 0.05 or less was deemed significant. This was a post-hoc analysis of the ARISTOTLE trial; sample size was determined by the availability of WSIs. For the UCLH cohort, the number of patients included was based on the availability of pre-treatment biopsies from LARC patients who received neoadjuvant chemoradiotherapy and were archived in the UCL/UCLH Biobank.

### Role of funders

The ARISTOTLE trial sample collection and slide scanning were funded by Cancer Research UK (A18745). The other funders played no direct roles in study design, data collection, data analyses, interpretation, or writing of the report. None of the authors received compensation from the funder or any other entity for writing this article.

## Results

### Patient characteristics

Table 2 shows the clinical characteristics and cell densities of the patients in ARISTOTLE-RC and UCLH-RC. The patient characteristics of TCGA-CRC are listed in Table S7.

**Table 2:** Patient characteristics in the two cohorts.

Characteristic	ARISTOTLE-RC (N = 414)	UCLH-RC (N = 70)
Age		
≤60	207 (50.0%)	21 (30.0%)
>60	207 (50.0%)	49 (70.0%)
Sex		
Female	140 (33.8%)	28 (40.0%)
Male	274 (66.2%)	42 (60.0%)
Treatment		
CRT	213 (51.4%)	70 (100.0%)
IrCRT	201 (48.6%)	0 (0.0%)
MRI T stage^a^		
T2	24 (5.8%)	14 (20.0%)
T3	321 (77.5%)	43 (61.4%)
T4	64 (15.5%)	13 (18.6%)
Missing	5 (1.2%)	0 (0.0%)
MRI N stage^a^		
N0	104 (25.1%)	17 (24.3%)
N1	184 (44.4%)	31 (44.3%)
N2	121 (29.2%)	22 (31.4%)
Missing	5 (1.2%)	0 (0.0%)
Post-Operation Pathological T stage	68 (16.4%)	3 (4.3%)
ypT0	12 (2.9%)	0 (0.0%)
ypT1	96 (23.2%)	9 (12.9%)
ypT2	163 (39.4%)	24 (34.3%)
ypT3	15 (3.6%)	5 (7.1%)
ypT4	60 (14.5%)	29 (41.4%)
Missing		
Post-Operation Pathological N stage^b^		
ypN0	244 (58.9%)	27 (38.6%)
ypN1	79 (19.1%)	10 (14.3%)
ypN2	30 (7.2%)	3 (4.3%)
ypNx	0 (0.0%)	1 (1.4%)
Missing	61 (14.7%)	29 (41.4%)
Median DFS (months)	51.8	33.6
Median OS (months)	60.2	47.0
5-year disease-free rate	75.5%	62.0%
5-year survival rate	61.2%	64.8%
5-year censoring rate	20.5%	33.8%
TIL density (mean ± std)	214.01 ± 204.26	271.43 ± 293.84
TAM density (mean ± std)	212.26 ± 173.46	229.72 ± 248.50
MI (mean ± std)	17.43 ± 13.75	15.48 ± 11.23

a Baseline MRI staging performed before the start of nCRT.

b “Nx” indicates cases where nodal status could not be assessed (e.g., insufficient pathological data or further evaluation required). “N missing” denotes cases where nodal information was not available due to missing imaging or clinical documentation.

### Prognostic value of immune cell infiltration and mitotic index

**Fig. 2:**
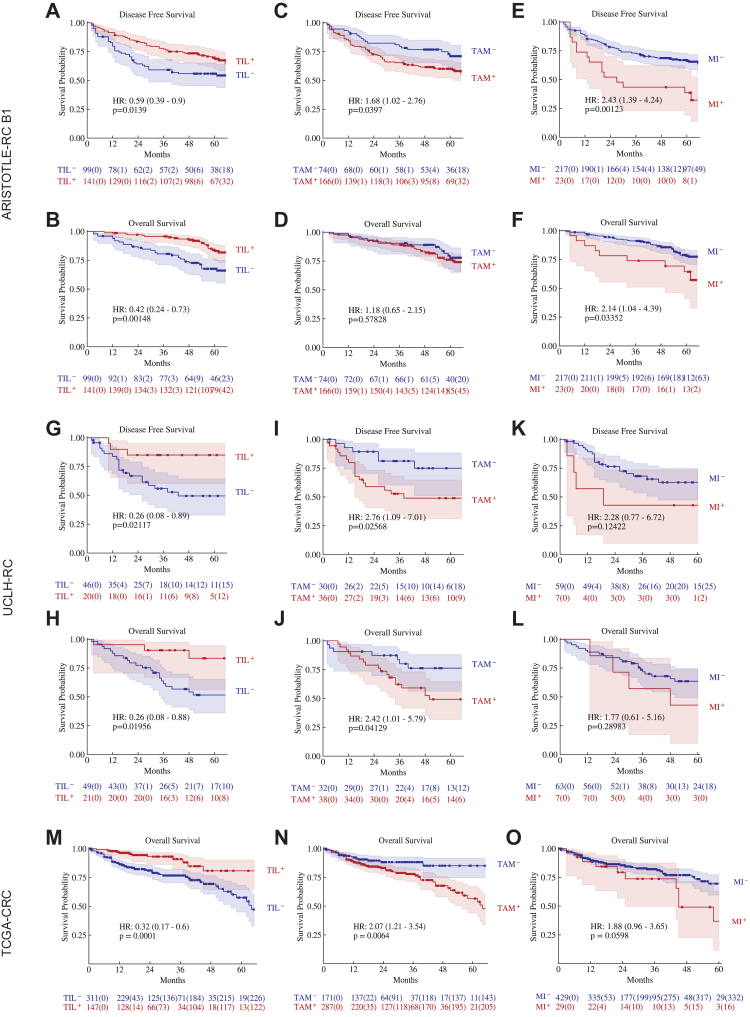
**The Kaplan–Meier curves of DFS and OS of the patients stratified by TIL, TAM density and MI in the ARISTOTLE-RC B1 (A–K), UCLH-RC (G–L) and TCGA-CRC (M–O).** TIL^+/−^, TAM^+/−^ and MI^+/−^ represent the patients with high (+)/low (−) levels of TIL, TAM densities and MI, respectively. p-values are from log-rank tests. The number of patients at risk in each subgroup is shown below the curves and the number of censored patients is indicated in parentheses.

In ARISTOTLE-RC B1, TIL^+^ patients had significantly better DFS (HR = 0.59, 95% CI: 0.39–0.9, p = 0.0139) and OS (HR = 0.42, 95% CI: 0.24–0.73, p = 0.00148) compared with TIL^−^ patients (Fig. 2A and B). The TAM^+^ patients showed significantly shorter DFS (HR = 1.68, 95% CI: 1.02–2.76, p = 0.0397) compared with TAM^−^ patients (Fig. 2C). However, the difference in OS is not significant (Fig. 1D). An inverse association between TAM and TIL levels (χ^2^ = 8.10, p = 0.0044) was found. The MI^+^ patients had significantly shorter DFS (HR = 2.43, 95% CI: 1.39–4.24, p = 0.0012) and OS (HR = 0.42, 95% CI: 0.24–0.73, p = 0.0014) (Fig. 2E and F). Those patterns existed in both the SCRT and ICRT arms (Figures S4–S6).

In the UCLH-RC cohort, TIL^+^ patients had significantly better DFS (HR = 0.26, 95% CI: 0.08–0.89, p = 0.0212) and OS (HR = 0.26, 95% CI: 0.08–0.88, p = 0.0196) (Fig. 2G and H), while TAM^+^ patients had significantly shorter DFS (HR = 2.42, 95% CI: 1.01–5.79, p = 0.0413) and OS (HR = 2.76, 95% CI: 1.09–7.01, p = 0.0257) (Fig. 2I and J). TAM^+^ patients are also more likely to exhibit TIL^−^ (χ^2^ = 5.62, p = 0.0177). Since no patients were stratified as MI^+^ by the original cut-off value, we designate patients whose mitotic index falls within the top 10% as MI^+^, aligning with the percentage observed in the ARISTOTLE-RC cohort. The MI^+^ patients tended to have shorter DFS (HR = 2.28, 95% CI: 0.77–6.72, p = 0.1242) (Fig. 2K and L). The prognostic impact of TIL and TAM in the combined ARISTOTLE-RC and UCLH-RC cohort is presented in Table S9.

In the TCGA-CRC, the TIL^+^ patients also showed significantly longer OS (HR = 0.32, 95% CI: 0.17–0.59, p = 0.0001, Fig. 2M). The TAM^+^ had significantly shorter OS (HR = 2.16, 95% CI: 1.25–3.73, p = 0.0048) (Fig. 2N). A negative association between TAM and TIL status was also found (χ^2^ = 22.04, p < 0.0001). The MI^+^ patients tended to have shorter OS (HR = 1.88, 95% CI: 0.96–3.65, p = 0.0598, Fig. 2O).

### Composite stratification of immune cell infiltration and mutation status

**Fig. 3:**
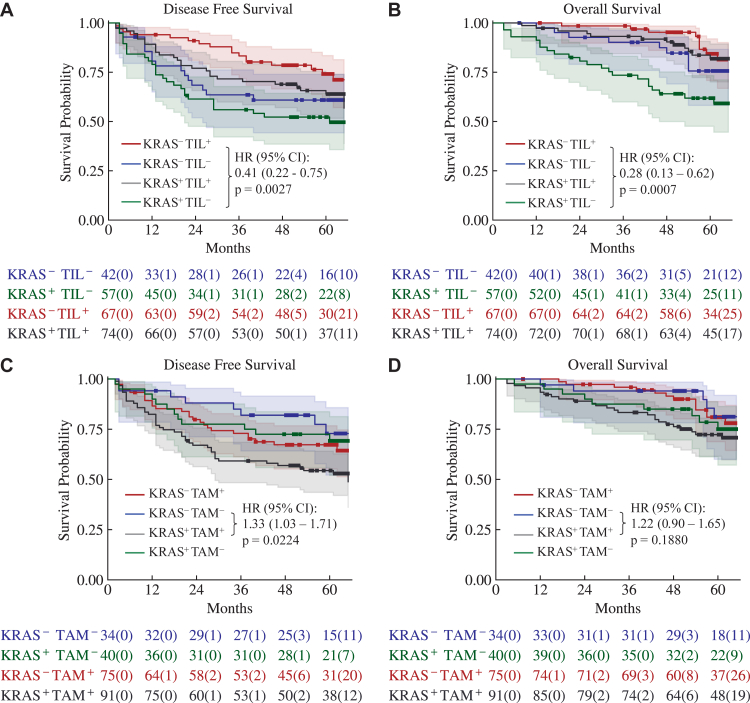
**The Kaplan–Meier curves of DFS and OS of the four subgroups stratified by *KRAS* mutation and TIL/TAM density in the ARISTOTLE-RC B1.** A. DFS of the subgroups stratified by KRAS and TIL. B. OS of the subgroups stratified by KRAS and TIL. C. DFS of the subgroups stratified by KRAS and TAM. D. OS of the subgroups stratified by KRAS and TAM. p-values are from log-rank tests. The number of patients at risk in each subgroup is shown below the curves and the number of censored patients is indicated in parentheses.

In ARISTOTLE-RC, *KRAS*^+^ patients tended to have shorter DFS (HR = 1.45, 95% CI: 0.94–2.23, p = 0.089) and OS (HR = 1.63, 95% CI: 0.92–2.87, p = 0.084) (Figure S7). TIL^+^ and *KRAS*^−^ patients had significantly longer DFS (HR = 0.41, 95% CI: 0.22–0.75, p = 0.0027) and OS (HR = 0.28, 95% CI: 0.13–0.62, p = 0.0007), compared to the TIL^−^ and *KRAS*^+^ group (Fig. 3A and B). TAM^+^ and *KRAS*^+^ patients had significantly shorter DFS (HR = 1.33, 95% CI: 1.03–1.71, p = 0.0224), compared to the TAM^−^ and *KRAS*^−^ group (Fig. 3C). No significant association was observed between *KRAS* mutation status and either TIL density (median difference = 10.15, 95% CI: −95.77 to 93.78, p = 0.8842) or TAM density (median difference = −143.26, 95% CI: −393.96 to 133.75, p = 0.5658).

In the UCLH-RC, the *KRAS* mutation status is only available for 18/70 patients and does not significantly differ in DFS (HR = 2.31, 95% CI: 0.61–8.68, p = 0.2039) and OS (HR = 1.33, 95% CI: 0.36–4.94, p = 0.6656) (Figure S7). No death or disease progression is observed in TIL^+^ and *KRAS*^−^ patients (Figure S8). TAM^+^ and *KRAS*^+^ patients had significantly shorter DFS (HR = 1.87, 95% CI: 0.92–3.78, p = 0.0494), compared to the TAM^−^ and *KRAS*^−^ group (Figure S8). In the combined cohort comprising ARISTOTLE-RC B1 and 18 patients from UCLH-RC with known *KRAS* mutation status, TIL^+^ and *KRAS*^−^ patients had significantly longer DFS (HR = 0.32, 95% CI: (0.18–0.58), p < 0.0001) and OS (HR = 0.23, 95% CI: 0.11–0.48, p p < 0.0001), compared to the TIL^−^ and *KRAS*^+^ group (Figure S9a and b). TAM^+^ and *KRAS*^+^ patients had significantly shorter DFS (HR = 1.37, 95% CI: 1.08–1.74, p = 0.0066) and tended to have shorter OS (HR = 1.26, 95% CI: 0.96–1.66, p = 0.0920), compared to the TAM^−^ and *KRAS*^−^ group (Figure S9C and D).

**Fig. 4:**
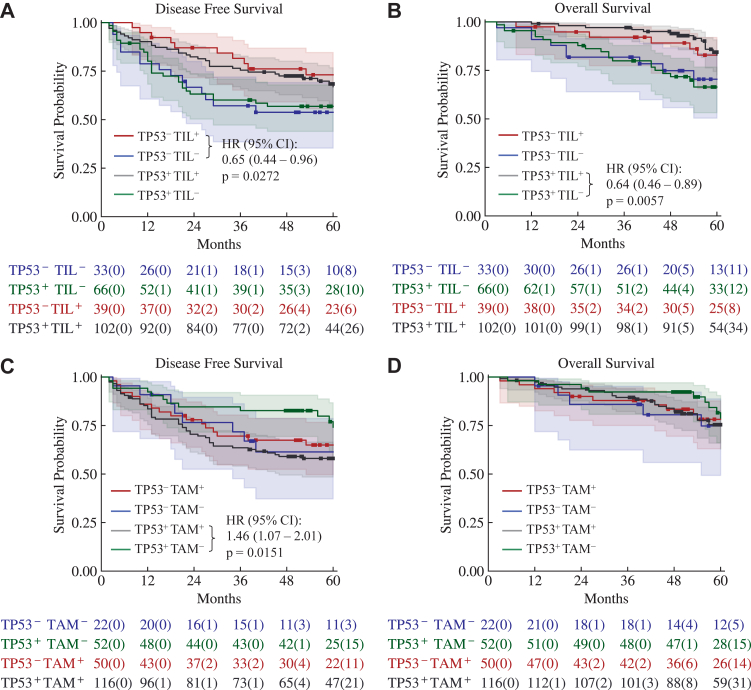
**The Kaplan–Meier curves of DFS and OS of the four subgroups stratified by *TP53* mutation and TIL/TAM density in the ARISTOTLE-RC B1.** A. DFS of the subgroups stratified by TP53 and TIL. B. OS of the subgroups stratified by TP53 and TIL. C. DFS of the subgroups stratified by TP53 and TAM. D. OS of the subgroups stratified by TP53 and TAM. p-values are from log-rank tests. The number of patients at risk in each subgroup is shown below the curves and the number of censored patients is indicated in parentheses.

We also assessed the prognostic impact of TIL and TAM in the context of *TP53* mutation status in ARISTOTLE-RC. No significant difference in DFS (HR = 1.02, 95% CI: 0.64–1.61, p = 0.9363) or OS (HR = 0.87, 95% CI: 0.49–1.55, p = 0.6368) was observed between *TP53*^-^ and *TP53*^+^ patients (Figure S10). No significant association was found between *TP53* mutation status and either TIL density (median difference = 63.19, 95% CI: −21.67 to 167.83, p = 0.2640) or TAM density (median difference = 15.11, 95% CI: −348.63 to 230.74, p = 0.9701). *TP53*^−^ and TIL^+^ had significantly improved DFS (HR = 0.65, 95% CI: 0.44–0.96, p = 0.0272) and tended to have longer OS (HR = 0.66, 95% CI: 0.40–1.10, p = 0.0991), compared to *TP53*^−^ and TIL^−^ patients. *TP53*^+^ and TIL^+^ patients tended to have longer DFS (HR = 0.83, 95% CI: 0.64–1.06, p = 0.1355) and had significantly longer OS (HR = 0.64, 95% CI: 0.46–0.89, p = 0.0057), compared to *TP53*^+^ and TIL^−^ patients (Fig. 4A and B). Notably, *TP53*^+^ and TAM^+^ patients showed significantly shorter DFS (HR = 1.46, 95% CI: 1.07–2.01, p = 0.0151), compared to *TP53*^+^ and TAM^−^ patients. In contrast, among *TP53* wild-type patients, no difference in DFS (HR = 1.00, 95% CI: 0.66–1.52, p = 0.9930) was observed between TAM^+^ and TAM^−^ subgroups (Fig. 4C). No significant difference in OS was observed across four subgroups (Fig. 4D). *TP53* mutation status is not available in UCLH-RC.

### Multivariable cox regression and added predictive value of TIME markers

Table 3 present the results of the multivariable Cox regression models incorporating TIL, TAM, and other pre-treatment baseline clinical variables. An additional analysis incorporating postoperative pathological stage, tumour location, and histological differentiation is presented in Table S8, although including pathological stage violated the proportional hazards assumption.

**Table 3:** Multivariable cox regression results with baseline clinical variables.

Variable	ARISTOTLE-RC		UCLH-RC		Combined Cohort	
	DFS (HR [95% CI))	OS (HR [95% CI))	DFS (HR [95% CI))	OS (HR [95% CI))	DFS (HR [95% CI))	OS (HR [95% CI))
Age (>60)	1.33 (0.85–2.09) p = 0.2144	2.09 (1.13–3.84) p = 0.0182	0.41 (0.12–1.40) p = 0.1558	1.79 (0.57–5.65) p = 0.3208	1.21 (0.82–1.79) p = 0.3397	2.02 (1.23–3.31) p = 0.0052
Sex (Male)	1.06 (0.66–1.71) p = 0.8060	0.70 (0.38–1.28) p = 0.2451	2.31 (0.82–6.50) p = 0.1120	0.94 (0.38–2.31) p = 0.8961	1.16 (0.77–1.75) p = 0.4800	0.79 (0.49–1.27) p = 0.3235
MRI T stage^a^						
T3	1.29 (0.46–3.64) p = 0.6336	0.96 (0.28–3.25) p = 0.9481	0.20 (0.03–1.19) p = 0.0760	1.47 (0.30–7.34) p = 0.6363	0.99 (0.46–2.13) p = 0.9723	0.97 (0.40–2.37) p = 0.9521
T4	1.99 (0.64–6.19) p = 0.2369	1.11 (0.28–4.37) p = 0.8763	0.70 (0.11–4.32) p = 0.6968	1.93 (0.39–9.45) p = 0.4185	1.65 (0.70–3.91) p = 0.2560	1.33 (0.49–3.65) p = 0.5758
MRI N stage^a^						
N1	0.75 (0.42–1.34) p = 0.3336	0.88 (0.40–1.95) p = 0.7552	0.96 (0.26–3.58) p = 0.9507	0.88 (0.29–2.64) p = 0.8134	0.76 (0.46–1.26) p = 0.2904	0.91 (0.49–1.68) p = 0.7632
N2	0.73 (0.39–1.37) p = 0.3302	1.05 (0.45–2.45) p = 0.9172	0.74 (0.16–3.48) p = 0.7079	0.71 (0.21–2.44) p = 0.5901	0.68 (0.39–1.18) p = 0.1737	0.92 (0.47–1.78) p = 0.8029
MRI circumferential Resection Margin (Involved)^a^	0.55 (0.27–1.10) p = 0.0904	0.70 (0.27–1.79) p = 0.4499	1.78 (0.57–5.52) p = 0.3180	2.03 (0.66–6.30) p = 0.2191	0.94 (0.53–1.67) p = 0.8253	1.03 (0.52–2.04) p = 0.9289
MRI extramural vascular invasion (Present)^a^	0.74 (0.24–2.26) p = 0.5935	2.23 (0.59–8.42) p = 0.2370	–	–	–	–
MRI extramural venous invasion (Present)^a^	3.05 (1.00–9.27) p = 0.0498	1.16 (0.31–4.29) p = 0.8250	8.13 (1.83–36.16) p = 0.0059	1.47 (0.48–4.50) p = 0.4980	2.31 (1.53–3.48) p = 0.0001	2.07 (1.26–3.40) p = 0.0039
*KRAS* (mutated)	1.33 (0.84–2.11) p = 0.2189	1.52 (0.83–2.77) p = 0.1742	–	–	–	–
*TP53* (mutated)	1.29 (0.79–2.09) p = 0.3060	1.10 (0.59–2.07) p = 0.7552	–	–	–	–
TIL (high)	0.60 (0.39–0.92) p = 0.0204	0.41 (0.23–0.73) p = 0.0026	0.97 (0.23–4.10) p = 0.9706	0.48 (0.12–1.93) p = 0.3042	0.59 (0.40–0.87) p = 0.0076	0.37 (0.23–0.61) p = 0.0001
TAM (high)	1.47 (0.87–2.48) p = 0.1487	0.86 (0.46–1.62) p = 0.6462	3.60 (1.17–11.09) p = 0.0258	1.77 (0.66–4.77) p = 0.2583	1.55 (0.99–2.43) p = 0.0576	1.00 (0.60–1.66) p = 0.9915
MI (high)	2.83 (1.57–5.11) p = 0.0005	2.65 (1.23–5.73) p = 0.0132	0.71 (0.16–3.11) p = 0.6543	1.76 (0.49–6.36) p = 0.3900	2.69 (1.62–4.49) p = 0.0001	2.45 (1.33–4.52) p = 0.0040

a From the baseline MRI performed before the start of nCRT.

Table 4 shows that the C-index derived from 10-fold cross-validation improved for both disease-free survival (DFS) and overall survival (OS) upon inclusion of the AI-identified TIME markers (TIL, TAM, and MI) in the Cox regression model. A likelihood ratio test (LRT) was also conducted to compare the full Cox model to a reduced model without the TIME markers. The results demonstrated that inclusion of these variables significantly improved model fit for both DFS (LRT χ^2^ = 24.81, p < 0.0001) and OS (LRT χ^2^ = 22.46, p < 0.0001).

**Table 4:** C-index of cox regression models with versus without TIME markers.

Model	DFS (C-index [95% CI])	OS (C-index [95% CI])	DFS (log-likelihood)	OS (log-likelihood)
Baseline clinical variables^a^	0.60 (0.52–0.68)	0.60 (0.54–0.67)	−591.73	−413.21
Baseline clinical variables + TIL, TAM and MI	0.65 (0.59–0.71)	0.66 (0.61–0.70)	−579.33	−401.98

a Clinical Variables presented in Table 3.

### Characterising dynamics of immune cell density after nCRT

**Fig. 5:**
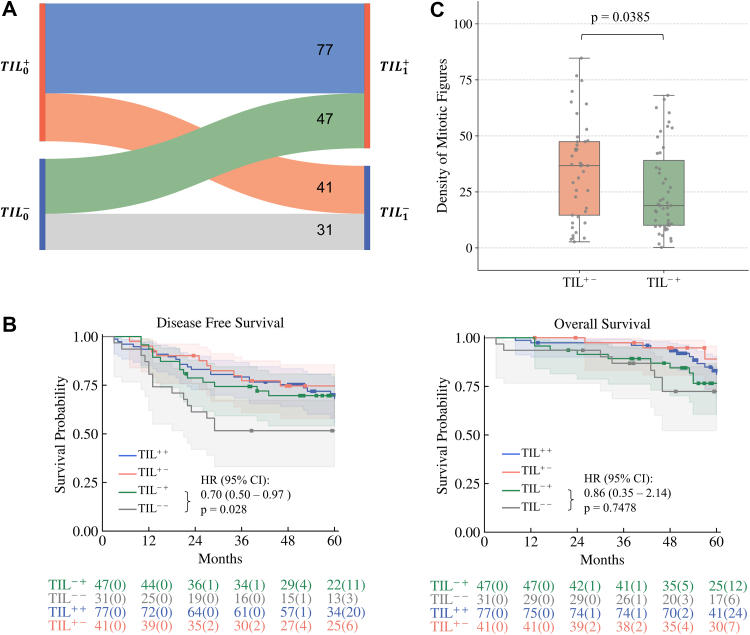
**The patient re-stratification after the nCRT.** A. The four re-stratification routes and the numbers in each group; B. The Kaplan–Meier curves of DFS (left) and OS (right) of TIL^++^, TIL^+−^, TIL^−+^ and TIL^−−^. p-values are from log-rank tests. The number of patients at risk in each subgroup is shown below the curves and the number of censored patients is indicated in parentheses. C. The density of mitotic figures in the TIL^+−^ and TIL^−+^ groups. The densities are presented using box plots with individual data points (N = 41 [left] and 47 [right] independent patients). p-value is from the Mann–Whitney U test.

In ARISTOTLE-RC B2, 89 cases showed re-stratification after the treatment, while 31 patients had persistently low immune infiltration (TIL^−−^, Fig. 5A) and exhibited a significantly shorter DFS. Patients undergoing treatment-related immune activation (TIL^− +^) appeared to have significantly improved DFS (HR = 0.70, 95% CI: 0.50–0.97, p = 0.0280), compared to the TIL^−−^ patients (Fig. 5B). Patients who experienced treatment-related immune suppression (TIL^+−^) also exhibited a significantly higher density of mitotic figures compared to the patient who experienced treatment-related immune activation (TIL^− +^) (median difference = 9.36, 95% CI: 1.87–16.85, p = 0.0385, Fig. 5C).

The TAM density also changes dynamically after the treatment, with 44 TAM^−+^ patients and 23 TAM^+−^ patients (Figure S11). Significantly shorter DFS was observed in the 121 TAM^++^patients (HR = 6.62, 95% CI: 1.02–48.14, p = 0.0302), compared to the 14 TAM^−−^ patients. TAM^+−^ patients also tended to have a higher density of mitotic figures, while not as significant (median difference = 6.96, 95% CI: 1.99–15.92, p = 0.2272).

## Discussion

We developed an AI framework for quantitatively analysing the tumour immune landscape and mitotic activity in digitised H&E-stained WSIs. The framework consists of a deep-learning-based image classifier to first identify the tumour-associated regions by tissue classification and a deep-learning-based object detector to detect the lymphocytes, macrophages, and mitotic figures. These measurements are used to calculate the density of TIL, TAM and MI. We applied this model to three CRC cohorts to stratify patients based on these features. Using the TCGA-CRC, we confirmed that our AI-derived TIL and TAM densities were associated with patient survival in a manner consistent with previous studies derived from the same cohort. Moreover, we specifically explored the prognostic relevance and post-treatment dynamics of TIME metrics and their interaction with DNA mutations in LARC patients following nCRT using a clinical trial cohort (ARISTOTLE-RC) and a real-world hospital cohort (UCLH-RC). To promote accessibility and clinical translation, we have developed a web-based platform (https://octopath.ai/) for automatically generating TIME metrics from uploaded slides.

### The prognostic impact of TIL and TAM

The AI-driven immune profiling reveals that both ARISTOTLE and UCLH cohorts displayed a strong association between high pre-treatment TIL density and longer survival in both the univariable and multivariable analyses. The prognostic impact of TIL has been widely reported. High TIL density is associated with longer DFS and OS in CRC patients with primary stage,^16^ including LARC.^50^^,^^51^ However, our study did not separate lymphocyte subtypes, for which the impact of their proportions, especially the cytotoxic T-cells, needs to be further investigated.

Macrophage infiltration has emerged as a research hotspot in recent years due to its potential role in tumour prognosis; however, studies in CRC have reported controversial findings.^18^, ^19^, ^20^, ^21^, ^22^, ^23^ Notably, although only a limited number of studies have focused exclusively on rectal cancer cohorts, all of them observed a negative prognostic impact.^25^^,^^26^^,^^52^ One possible explanation is that CRT is commonly used in patients with rectal cancer, yet no study has specifically shown the impact of TAM in CRT-treated cohorts. In this study, similar results were found in the two CRT-treated rectal cancer cohorts, where high TAM density is associated with worse DFS in the ARISTOTLE-RC and OS in the UCLH-RC. The association between TAM and DFS remains significant in the multivariable analysis, indicating the predictive value of TAM on disease progression. We also found that TAM^+^ patients had worse OS in the TCGA-CRC, which is consistent with the previous study.^24^ Future analyses should include rectal cancer cohorts not treated with CRT to better understand the influence of CRT on TAM behaviour. High infiltration of M1-like macrophages is generally associated with improved survival in CRC, while M2-like macrophages tend to promote tumour progression and are linked to worse outcomes.^53^^,^^54^ However, the AI framework used in this study detects aggregated macrophages. Future studies are needed to investigate the prognostic significance of specific TAM subtypes.

### Immune activity and DNA mutation

We found that *KRAS*-mutated patients with low TIL density experience significantly worse outcomes in both DFS and OS, compared to *KRAS*-wildtype patients with high TIL density. While high TIL density has already been established as a favourable prognostic factor, the *KRAS* mutation provides an additive prognostic value. Similarly, *KRAS* mutation status and TAM levels also demonstrated an additive impact on prognosis. *KRAS* mutations occur in approximately 30%–50% of CRC patients and have been associated with poor clinical outcomes.^55^ However, few studies have explored the co-impact of the *KRAS* mutation and TIL density in CRC^56^^,^^57^ and data on DFS and OS stratified by both TIL density and *KRAS* status remain scarce. A study by Liu, H. et al. indicated that the impact of TAMs on OS is more significant in *KRAS*-mutated tumours.^58^

Studies reporting the association between *KRAS* mutation and immune cell densities in CRC are also limited. Fu, X. et al. reported a potential association between *KRAS* mutations and lower TIL density,^59^ while Mojarad, E. et al. observed a correlation between *KRAS* mutations and stromal lymphocyte density, rather than intra-tumoral TILs.^57^ Liu, H. et al. found a positive correlation between *KRAS* mutation and TAM expression.^58^ However, in our cohorts, neither TAM nor TIL densities were associated with *KRAS* mutation status. Further studies are required to investigate the interactions between *KRAS* mutations and immune activity within TIME.

We also found that high TAM density was associated with significantly worse DFS specifically in *TP53*-mutated patients. This observation can be explained by the findings of Cooks et al.,^60^ who reported that mutant p53 proteins, particularly those with gain-of-function (GOF) mutations, can reprogramme macrophages toward a tumour-promoting phenotype. Although our study did not include molecular subtyping of *TP53* mutations (GOF versus non-GOF), we still observed a differential impact of TAM density in *TP53*-mutated versus *TP53*-wildtype subgroups. This supports the hypothesis that *TP53* mutation status may modulate macrophage behaviour, and the combination of *TP53* and the AI-identified TAM could serve as risk stratification factors for LARC patients undergoing nCRT.

The absence of multi-omics data in the UCLH-RC cohort restricts the ability to independently validate molecular and immune-related findings derived from the ARISTOTLE-RC cohort. Future work will involve whole-genome sequencing and transcriptomic profiling of retrospective samples for comprehensive validation.

### Immune activity, CRT and tumour proliferation

By comparing the TIL density before and after nCRT, we observed that patients with consistently pre-/post-treatment low TIL levels (TIL^−−^) had the poorest outcomes. Noticeably, TIL^−+^ patients, who showed increased immune activity post-treatment, demonstrated significantly improved DFS. No significant difference was found between the TIL^++^ and TIL^+−^ patients, suggesting that patients with an active immune response before the treatment tend to have better outcomes, even if it is suppressed by the treatment. While only 14 patients exhibited consistently low TAM density (TAM^−−^), this group had significantly longer DFS and tended to have better OS, with no deaths recorded.

Several studies have reported that nCRT could activate anti-tumour immunity by increasing the number of CD8^+^ TILs, which is associated with better outcomes.^61^, ^62^, ^63^ The CRT-induced immune activity is also related to microsatellite instability (MSI) status, methylation level and HLA class I expression.^64^, ^65^, ^66^ However, all the patients in ARISTOTLE-RC B2 are microsatellite-stable, and only 1% of them show HLA class I expression, leaving us unable to validate those findings in this study. No association was found between the change in TIL and TAM density and methylation value or the DNA mutations of 80 genes (Table S10).

Of note, we found that patients with increased TIL post-treatment had significantly higher mitotic index (MI) compared to those with decreased TIL. A similar pattern was seen in TAM dynamics, with higher mean MI in TAM^+−^ patients than in TAM^−+^ patients. High tumour proliferative activity may suppress immune responses after nCRT. Specht, J. reported increased TIL and TAM infiltration in breast cancer with G2/M arrest.^67^ However, further research is needed to understand how tumour proliferation interacts with immune activation in CRC.

MI itself appeared prognostic in this study. MI^+^ patients had significantly shorter DFS and OS in ARISTOTLE-RC and tended toward shorter OS in TCGA-CRC. A limited number of studies have reported the impact of MI on CRC. The study from Li, B. et al. reported that the cell mitosis was not associated with tumour regression grade in 106 CRC patients after nCRT, while long-term survival was not compared.^68^ Notably, the threshold used to define MI^+^ status in our study was relatively high, resulting in only 10% and 6% of patients being classified as MI^+^ in the ARISTOTLE-RC and TCGA-CRC cohorts, respectively. Furthermore, no MI^+^ cases were observed in the UCLH-RC cohort, likely due to the small sample size and the staining and scanning variability across institutions. We have recalibrated the cut-off for the UCLH-RC cohort, designating as MI^+^ those patients whose mitotic index falls within the top 10%, mirroring the proportion used in the ARISTOTLE-RC cohort. However, recalibration may not be ideal as this assumes that mitotic activity distributions should be directly comparable despite differences in sample size, tumour biology, or technical factors. Future studies with larger cohorts are planned to further evaluate the prognostic role of tumour mitosis.

### Advantages and disadvantages of AI-powered TIME analysis

Manual quantification of immune cells within TIME is labour-intensive, subject to large intra-observer variability, and time-consuming, posing limitations for large-scale studies.^69^^,^^70^ In contrast, AI models offer rapid, consistent, and scalable analysis. Additionally, AI-based approaches improve robustness and reproducibility, addressing the variability often observed among pathologists in grading immune cell infiltration or assessing mitotic activity.

Noticeably, the AI-identified macrophage densities in this study were lower than commonly reported values in the literature.^71^ This discrepancy can be attributed to two main factors: we computed average densities across the entire tumour and tumour-associated stroma, rather than hotspot regions or the invasive margin, which typically show higher localised macrophage infiltration; many studies used tissue microarrays or selected regions of interest,^72^ while our whole-slide analysis captures both high- and low-density areas, resulting in a more conservative overall estimate.

Distinguishing immune cell subtypes from H&E-stained WSIs is challenging, which restricts the analysis of subtype-specific immune markers. In other studies, specific TIL subsets, such as cytotoxic, helper, and regulatory T cells, have been identified using immunohistochemical staining with markers including CD8, CD4, and FOXP3, respectively.^16^ Similarly, efforts have been made to classify M1/M2-like macrophages using molecular markers.^18^ Commonly reported markers include CD80, CD86, and iNOS for M1-like macrophages, and CD163, CD206, and Arginase-1 for M2-like macrophages.^73^ However, macrophage subtyping remains inherently challenging due to their remarkable plasticity and the existence of a spectrum of activation states, rather than discrete subsets.^74^

Despite this, numerous studies have demonstrated that aggregate immune cell density, particularly total TAM burden, retains prognostic and predictive value even without subclassification.^75^ Additionally, total TAM density has also been linked to response to immune checkpoint blockade in various cancers,^76^ highlighting the clinical significance of quantifying general TAM density. Moreover, our approach offers a practical and clinically scalable solution for profiling the TIME, especially in resource-limited settings or retrospective cohorts where advanced multiplexed assays are not available. In future studies, we plan to integrate AI with subtype-specific techniques such as multiplex fluorescence imaging or spatial transcriptomics.

The quality control (QC) process in this study relied on manual review, which, while effective for the two LRAC cohorts, is insufficient for large-scale cohorts such as TCGA. Although staining and spatial augmentations were applied during model development to enhance generalisability, the lack of QC in TCGA-CRC may still introduce bias in AI-driven quantification. Scalable automated QC pipelines will be incorporated in future studies. Another limitation is that pre-treatment biopsies may not capture the full extent of tumour heterogeneity, particularly in terms of immune infiltration and spatial architecture. However, pre-treatment pathological assessment typically relies on biopsies, as post-surgical resections are often altered by neoadjuvant therapies, making them less suitable for baseline biomarker evaluation. Future studies will aim to use multiple biopsies from different tumour regions.

In conclusion, this study investigated the prognostic impact of AI-identified immune cell densities and mitotic index in three independent cohorts, comprising one all-stage CRC cohort and two LARC cohorts treated with nCRT. We found that high TIL density and low TAM density were associated with improved DFS and OS, and their impact was modulated by DNA mutation status. Additionally, patients whose TIL density increased following nCRT had improved DFS. A potential link between tumour proliferation and immune suppression was also discussed. These findings support the integration of immune and proliferative biomarkers into future LARC risk stratification and nCRT treatment planning.

Future work will focus on external validation and clinical translation. To support that, we have developed a web-based platform (https://octopath.ai/) that enables users to upload H&E-stained whole-slide images and apply our AI models for TIME assessment. A prospective clinical study to evaluate the real-world impact of this tool in routine pathology workflows is planned. In parallel, we aim to apply our models to immunotherapy-treated cohorts to assess whether AI-identified TIME features from H&E slides can stratify patients by treatment response and identify those most likely to benefit from immunotherapy.

## Contributors

CACF, MAH and ZS designed the study, with input from the ARISTOTLE trial team (DS, AL, RB). ZS, MS and CACF developed the AI framework. ZS analysed the data and wrote the draft manuscript. DB, SH, MAH contributed to the clinical interpretation. NW provided the digital pathology data of the ARISTOTLE trial cohort. TSM provided the NGS data of the ARISTOTLE trial cohort. APL, TM, DO provided the digital pathology data of the UCLH-RC cohort. All the authors contributed to the manuscript revision and were involved in the scientific discussion. All authors read and approved the final version of the manuscript. ZS, CACF, MAH, DB had full access to and verified the integrity of the underlying data.

## Data sharing statement

The clinical and pathology data of the trial participants are not publicly available due to restrictions by the ethical approval of this study. To get access to the ARISTOTLE dataset and the UCLH dataset, a signed data access agreement ensuring compliance with ethical guidelines, approval from the institutional review board, and submission of a detailed proposal outlining the intended use of the data will be needed. Requests will be considered on a case-by-case basis and should be directed to and the clinical trial unit (ctc.aristotle@ucl.ac.uk) and UCL/UCLH Biobank (ci.bbfhad-admin@ucl.ac.uk) at University College London. The TCGA data is available via Genomic Data Commons Data Portal (https://portal.gdc.cancer.gov/). The codes and parameters of the AI models are available on GitHub: https://github.com/cacof1/DigitalPathologyAI. The AI models for cell detection can be inferenced via https://octopath.ai/. The datasets used for developing the AI models are available on Zenodo: Tissue Classification: https://zenodo.org/records/1214456, Immune Cell Detection: https://zenodo.org/records/11073373 and Mitotic Figure Detection: https://zenodo.org/records/14246170. For any other requests and queries relating to data sharing, please contact the corresponding author zhuoyan.shen.18@ucl.ac.uk.

## Declaration of interests

TSM received a consulting fee from Nordic Pharma and was the Interim Chief Executive of the National Cancer Research Institute (NCRI) in 2022. NPW is supported by grants from Yorkshire Cancer Research, GSK, Pierre Fabre, Roche Diagnostics and NCRI, and received consulting fees from Bristol Myers, Squibb, Astellas, Pfizer, GSK, Amgen, Servier, and Beigene. He also has patents planned, issued, or pending with Roche Diagnostics. SH is supported by the Radiation Research Unit at the Cancer Research UK City of London Centre.
