# Supplementary material for: AI-powered immune profiling from histopathology slides for chemo-radiotherapy outcome prediction in rectal cancer: a study using clinical trial and real-world cohorts
Source: eBioMedicine. 2025 Nov 17;122:105993. doi: 10.1016/j.ebiom.2025.105993 (PMC12790592; doi:10.1016/j.ebiom.2025.105993)
Supplement: Supplementary Materials [file mmc1.docx]

**AI-Powered Immune Profiling from Histopathology Slides for Chemo-Radiotherapy Outcome Prediction in Rectal Cancer: A Study Using Clinical Trial and Real-World Cohorts**

Zhuoyan Shen*^1^, Douglas Brand^1,2^, Mikael Simard^1^, Adam P. Levine^3,4^, Sumeet Hindocha^1,2^, Talisa Mistry^3^, Dahmane Oukrif^3^, Andre Lopes^5^, Rubina Begum^5^, Nicholas P. West^6^, Ying Zhang^1^, Gary Royle^1^, Tim S. Maughan^7^, David Sebag-Montefiore^8^, Maria A. Hawkins^1,2^ and Charles-Antoine Collins Fekete^1^

1. Department of Medical Physics and Biomedical Engineering, University College London, London, UK
2. Department of Radiotherapy, University College London Hospitals NHS Foundation Trust, London, UK
3. Research Department of Pathology, University College London, London, UK
4. Department of Cellular Pathology, University College London Hospitals NHS Foundation Trust, London, UK
5. UCL Cancer Institute, London, UK.
6. Division of Pathology and Data Analytics, Leeds Institute of Medical Research, School of Medicine, University of Leeds, Leeds, UK
7. Department of Oncology, University of Oxford, Oxford, UK
8. Division of Oncology, Leeds Institute of Medical Research, School of Medicine, University of Leeds, Leeds, UK

**Supplementary Tables**

**Table S1. Information about the data used in each cohort.**

| **Cohort**  **(Year of Diagnosis)** | **Data type** | **Description** |
| --- | --- | --- |
| ARISTOTLE-RC  (2011-2018) | Clinical information | Age, sex, treatment arm, MRI stage, MRI circumferential resection margin, MRI extramural vascular invasion, MRI extramural venous invasion, pathological stage, tumour location, differentiation, post-operative adjuvant chemotherapy status, disease-free survival, overall survival |
|  | Digitised WSIs | Digitised WSIs of haematoxylin and eosin (H&E)-stained pre-treatment biopsy (N = 414) and post-treatment resection (N = 202) specimens obtained from formalin-fixed paraffin-embedded (FFPE) tissue samples. The detailed histopathology and quality assurance procedures are described in the ARISTOTLE trial protocol (Version 7.1, November 2022, available at <https://www.ctc.ucl.ac.uk/TrialDetails.aspx?Trial=82>). WSIs were sourced from the Virtual Pathology Database at the University of Leeds. All images were scanned using Leica Aperio GT 450 DX at 20× magnification, with a pixel size of 0.5 µm per pixel.  Prior to AI analysis, all WSIs were manually reviewed to exclude slides exhibiting significant artifacts and abnormal staining. |
|  | Mutation data | DNA mutation data of 80 colorectal cancer driver genes from next-generation sequencing. |
| UCLH-RC  (2013-2022) | Clinical information | Age, sex, MRI stage, MRI circumferential resection margin, MRI extramural venous invasion, pathological stage, tumour location, disease-free survival, overall survival |
|  | Digitised WSIs | Digitised WSIs of H&E-stained pre-treatment biopsies (N = 70) obtained from FFPE tissue samples. WSIs were generated at the Research Department of Pathology at University College London. Tissue processing followed routine UCLH Department of Pathology standard operating procedures, also performed under UKAS ISO 15189 accreditation. All images were scanned using Leica Aperio GT 450 DX at 40× magnification, with a pixel size of 0.26 µm per pixel.  Prior to AI analysis, all WSIs were manually reviewed to exclude slides exhibiting significant artifacts and abnormal staining. |
|  | Mutation data | KRAS mutation data from next-generation sequencing. |
| TCGA-CRC  (1998-2013) | Clinical information | Age, sex, site, pathological stage, overall survival |
|  | Digitised WSIs | Digitised WSIs of H&E-stained diagnostic specimens (N = 458) obtained from FFPE tissue samples. WSIs were downloaded from GDC Data Portal of The Cancer Genome Atlas Program (TCGA). All images were scanned at 40× magnification, with pixel sizes ranges from 0.24 - 0.26 µm per pixel. |

**Table S2. Comparison between the whole ARISTOTLE cohort (N = 589) and subgroups used in this study**

| **Cohort** | **Variable** | **Test** | **Statistic** | **p** |
| --- | --- | --- | --- | --- |
| ARISTOTLE-RC  (N = 414/589) | Age | T-test | -0.15 | 0.8841 |
|  | Sex | Chi-squared | 0.00 | 1.0000 |
|  | Treatment Arm | Chi-squared | 0.17 | 0.6786 |
|  | MRI T stage | Chi-squared | 0.36 | 0.9857 |
|  | MRI N stage | Chi-squared | 1.09 | 0.9549 |
|  | DFS | Log-rank test | 0.00 | 0.9906 |
|  | OS | Log-rank test | 0.03 | 0.8594 |
| ARISTOTLE-RC B1  (N = 240/589) | Age | T-test | -0.63 | 0.5283 |
|  | Sex | Chi-squared | 0.00 | 1.0000 |
|  | Treatment Arm | Chi-squared | 0.24 | 0.6243 |
|  | MRI T stage | Chi-squared | 0.98 | 0.9135 |
|  | MRI N stage | Chi-squared | 1.84 | 0.8709 |
|  | DFS | Log-rank test | 0.33 | 0.5646 |
|  | OS | Log-rank test | 0.47 | 0.4924 |
| ARISTOTLE-RC B2  (N = 202/589) | Age | T-test | -0.58 | 0.5595 |
|  | Sex | Chi-squared | 1.65 | 0.1989 |
|  | Treatment Arm | Chi-squared | 0.30 | 0.5847 |
|  | MRI T stage | Chi-squared | 1.41 | 0.8427 |
|  | MRI N stage | Chi-squared | 1.90 | 0.8633 |
|  | DFS | Log-rank test | 2.88 | 0.0898 |
|  | OS | Log-rank test | 2.92 | 0.0976 |

**Table S3. Datasets used for AI model development.**

| **Task** | **Model** | **Dataset** | **Data Size** | **Data Split** |
| --- | --- | --- | --- | --- |
| Tissue Classification | EfficientNet B0 | NCT-CRC-HE-100K | 107,180 tiles | A total of 10,000 tiles were used for training and validation, with 20% allocated to the validation set. An independent hold-out test set comprising 7,180 tiles was used for testing. |
| Immune Cell Detection | Yolov10 | Immunocto | 2,282,818 cells from 40 WSIs | The dataset was split on a WSI-wise basis with 35 WSIs used for training, 4 WSIs used for validation, and a holdout WSI used for testing. |
| Mitotic Figure Detection | Yolov10 | OMG-Octo | 74, 620 mitotic figures from 938 samples | The dataset was split on an image-wise basis with 661 samples used for training, 166 samples used for validation, and 111 samples used for testing. |

**Table S4. Augmentation Settings and Hyperparameters for YOLOv10 implemented using ultralytics.**

| Argument | Value | Description |
| --- | --- | --- |
| hsv_h | 0.015 | Adjusts the hue of the image by a fraction of the color wheel, introducing color variability. Helps the model generalize across different lighting conditions. |
| hsv_s | 0.2 | Alters the saturation of the image by a fraction, affecting the intensity of colors. Useful for simulating different environmental conditions. |
| hsv_v | 0.2 | Modifies the value (brightness) of the image by a fraction, helping the model to perform well under various lighting conditions. |
| degrees | 0.0 | Rotates the image randomly within the specified degree range, improving the model's ability to recognize objects at various orientations. |
| translate | 0.1 | Translates the image horizontally and vertically by a fraction of the image size, aiding in learning to detect partially visible objects. |
| scale | 0.0 | Scales the image by a gain factor, simulating objects at different distances from the camera. |
| shear | 0.0 | Shears the image by a specified degree, mimicking the effect of objects being viewed from different angles. |
| perspective | 0.0 | Applies a random perspective transformation to the image, enhancing the model's ability to understand objects in 3D space. |
| flipud | 0.0 | Flips the image upside down with the specified probability, increasing the data variability without affecting the object's characteristics. |
| fliplr | 0.5 | Flips the image left to right with the specified probability, useful for learning symmetrical objects and increasing dataset diversity. |
| bgr | 0.0 | Flips the image channels from RGB to BGR with the specified probability, useful for increasing robustness to incorrect channel ordering. |
| mosaic | 1.0 | Combines four training images into one, simulating different scene compositions and object interactions. Highly effective for complex scene understanding. |
| mixup | 0.0 | Blends two images and their labels, creating a composite image. Enhances the model's ability to generalize by introducing label noise and visual variability. |
| cutmix | 0.0 | Combines portions of two images, creating a partial blend while maintaining distinct regions. Enhances model robustness by creating occlusion scenarios. |
| copy_paste | 0.0 | Segmentation only. Copies and pastes objects across images to increase object instances. |
| copy_paste_mode | flip | Segmentation only. Specifies the copy-paste strategy to use. Options include 'flip' and 'mixup'. |
| auto_augment | randaugment | Classification only. Applies a predefined augmentation policy ('randaugment', 'autoaugment', or 'augmix') to enhance model performance through visual diversity. |
| erasing | 0.4 | Classification only. Randomly erases regions of the image during training to encourage the model to focus on less obvious features. |

**Table S5. Performance of the AI framework on detecting tissues and cells.**

| **Class** | **Validation Set** | | | **Testing Set** | | |
| --- | --- | --- | --- | --- | --- | --- |
|  | **Precision** | **Recall** | **F1 Score** | **Precision** | **Recall** | **F1 Score** |
| Tumour | 0.991 | 0.989 | 0.990 | 0.978 | 0.964 | 0.971 |
| Stroma | 0.887 | 0.834 | 0.860 | 0.840 | 0.798 | 0.818 |
| Lymphocyte | 0.780 | 0.701 | 0.738 | 0.792 | 0.723 | 0.756 |
| Macrophage | 0.733 | 0.528 | 0.614 | 0.750 | 0.532 | 0.622 |
| Mitotic Figure | 0.785 | 0.793 | 0.789 | 0.798 | 0.816 | 0.807 |

**Table S6. Cut-off values represented by cell densities per mm^2^**

| **Cut-off (cells per 2 mm^2^)** | **Cut-off (cells per mm^2^)** |
| --- | --- |
| 270.33 | 135.16 |
| 173.44 | 86.72 |
| 40.37 | 20.18 |

**Table S7. Patient characteristics of the TCGA_CRC**

| **Characteristic** | | **TCGA_CRC (N = 458)** |
| --- | --- | --- |
| Age | ≤ 60 | 133 |
|  | > 60 | 325 |
| Sex | Female | 190 |
|  | Male | 268 |
| Site | Colon | 396 |
|  | Rectal | 62 |
| Ethnicity | Hispanic or Latino | 5 |
|  | Not Hispanic or Latino | 252 |
|  | Not reported | 201 |
| AJCC Staging Edition | 2^nd^ | 1 |
|  | 5^th^ | 54 |
|  | 6^th^ | 167 |
|  | 7^th^ | 187 |
| Pathological T stage | T0 | 1 |
|  | T1 | 12 |
|  | T2 | 87 |
|  | T3 | 313 |
|  | T4 | 45 |
| Pathological N stage | N0 | 274 |
|  | N1 | 118 |
|  | N2 | 66 |
| Pathological M stage | M0 | 359 |
|  | M1 | 59 |
|  | Mx | 37 |
| Mean OS (months) | | 24.37 |
| 5-year survival rate | | 82.31% |
| 5-year censoring rate | | 5.24% |
| TIL density (mean ± std) | | 248.92 ± 231.28 |
| TAM density (mean ± std) | | 220.20 ± 148.94 |
| MI (mean ± std) | | 27.34 ± 10.77 |

**Table S8. Multivariable Cox regression results with post-treatment pathological variables.**

| **Variable** | **ARISTOTLE-RC** | | **UCLH-RC** | | **Combined Cohort** | |
| --- | --- | --- | --- | --- | --- | --- |
|  | DFS (HR [95%CI]) | OS (HR [95%CI]) | DFS (HR [95%CI]) | OS (HR [95%CI]) | DFS (HR [95%CI]) | OS (HR [95%CI]) |
| Age (>60) | 1.59 (0.99-2.56)  p = 0.0563 | 2.54 (1.36-4.76)  p = 0.0035 | 0.25 (0.06-1.01)  p = 0.0514 | 3.38 (0.87-13.10)  p = 0.0787 | 1.46 (0.96-2.21)  p = 0.0752 | 2.61 (1.52-4.46)  p = 0.0005 |
| Sex (Male) | 0.92 (0.56-1.52)  p = 0.7543 | 0.53 (0.28-1.00)  p = 0.0515 | 5.38 (1.37-21.10)  p = 0.0158 | 1.80 (0.62-5.22)  p = 0.2779 | 1.06 (0.69-1.61)  p = 0.7928 | 0.73 (0.45-1.19)  p = 0.2100 |
| MRI T stage* |  |  |  |  |  |  |
| T3 | 1.11 (0.38-3.26)  p = 0.8436 | 0.73 (0.20-2.69)  p = 0.6404 | 0.06 (0.01-0.62)  p = 0.0181 | 4.19 (0.57-30.74)  p = 0.1584 | 1.08 (0.49-2.37)  p = 0.8526 | 1.05 (0.41-2.66)  p = 0.9177 |
| T4 | 1.60 (0.48-5.34)  p = 0.4406 | 0.63 (0.14-2.77)  p = 0.5397 | 1.33 (0.12-14.31)  p = 0.8160 | 5.66 (0.70-45.60)  p = 0.1033 | 1.61 (0.65-4.02)  p = 0.3042 | 1.21 (0.41-3.52)  p = 0.7293 |
| MRI N stage* |  |  |  |  |  |  |
| N1 | 0.81 (0.44-1.49)  p = 0.4972 | 0.89 (0.38-2.09)  p = 0.7871 | 0.11 (0.02-0.67)  p = 0.0167 | 0.28 (0.07-1.18)  p = 0.0839 | 0.73 (0.44-1.24)  p = 0.2448 | 0.86 (0.45-1.62)  p = 0.6363 |
| N2 | 0.66 (0.34-1.28)  p = 0.2168 | 0.95 (0.38-2.37)  p = 0.9191 | 0.19 (0.03-1.22)  p = 0.0801 | 0.44 (0.12-1.70)  p = 0.2361 | 0.62 (0.34-1.10)  p = 0.1038 | 0.79 (0.39-1.61)  p = 0.5157 |
| MRI circumferential Resection Margin (Involved)* | 0.62 (0.30-1.28)  p = 0.1966 | 1.05 (0.39-2.87)  p = 0.9225 | 1.11 (0.25-4.91)  p = 0.8896 | 1.46 (0.43-4.91)  p = 0.5418 | 0.93 (0.52-1.67)  p = 0.8017 | 1.08 (0.53-2.16)  p = 0.8378 |
| MRI extramural vascular invasion (Present)* | 0.64 (0.19-2.13)  p = 0.4676 | 2.59 (0.68-9.85)  p = 0.1626 | - | - | - | - |
| MRI extramural venous invasion (Present)* | 2.98 (0.89-9.97)  p = 0.0763 | 0.81 (0.22-3.00)  p = 0.7565 | 132.45 (10.82-1620.94)  p = 0.0001 | 1.68 (0.48-5.85)  p = 0.4150 | 2.07 (1.36-3.16)  p = 0.0008 | 1.66 (0.99-2.77)  p = 0.0534 |
| KRAS (mutated) | 1.29 (0.80-2.09)  p = 0.2955 | 1.27 (0.67-2.41)  p = 0.4550 | - | - | - | - |
| TP53 (mutated) | 1.15 (0.69-1.91)  p = 0.5869 | 0.81 (0.41-1.59)  p = 0.5442 | - | - | - | - |
| Pathological T stage |  |  |  |  |  |  |
| pT2 | 0.30 (0.14-0.63)  p = 0.0015 | 0.20 (0.06-0.66)  p = 0.0077 | 198.15 (11.21-3503.49)  p = 0.0003 | 0.50 (0.07-3.49)  p = 0.4841 | 0.43 (0.23-0.83)  p = 0.0113 | 0.25 (0.10-0.64)  p = 0.0039 |
| pT3 | 0.83 (0.46-1.50)  p = 0.5390 | 1.99 (0.96-4.13)  p = 0.0662 | 18.86 (3.45-103.05)  p = 0.0007 | 0.91 (0.28-3.00)  p = 0.8813 | 0.98 (0.59-1.62)  p = 0.9232 | 1.24 (0.70-2.18)  p = 0.4665 |
| pT4 | 2.21 (0.85-5.74)  p = 0.1033 | 2.72 (0.67-10.98)  p = 0.1610 | 41.96 (2.77-635.94)  p = 0.0071 | 9.33 (1.41-61.61)  p = 0.0204 | 2.41 (1.12-5.16)  p = 0.0241 | 2.69 (1.09-6.64)  p = 0.0315 |
| Pathological N stage |  |  |  |  |  |  |
| N1 | 1.11 (0.59-2.11)  p = 0.7391 | 0.83 (0.36-1.93)  p = 0.6630 | 0.40 (0.03-5.13)  p = 0.4847 | 0.35 (0.05-2.36)  p = 0.2821 | 1.22 (0.72-2.08)  p = 0.4524 | 0.93 (0.48-1.80)  p = 0.8393 |
| N2 | 1.72 (0.68-4.37)  p = 0.2553 | 2.33 (0.73-7.42)  p = 0.1534 | 12.85 (0.69-240.35)  p = 0.0874 | 17.34 (1.01-296.95)  p = 0.0490 | 2.00 (0.88-4.53)  p = 0.0959 | 2.87 (1.09-7.58)  p = 0.0329 |
| Tumour Location |  |  |  |  |  |  |
| Middle | 0.63 (0.28-1.40)  p = 0.2549 | 0.30 (0.10-0.90)  p = 0.0321 | 0.24 (0.05-1.18)  p = 0.0798 | 3.27 (1.09-9.83)  p = 0.0347 | 0.71 (0.39-1.29)  p = 0.2613 | 0.97 (0.51-1.84)  p = 0.9254 |
| High | 0.22 (0.05-0.99)  p = 0.0482 | 0.17 (0.02-1.37)  p = 0.0961 | 0.18 (0.03-0.93)  p = 0.0406 | 0.72 (0.18-2.88)  p = 0.6375 | 0.37 (0.15-0.89)  p = 0.0270 | 0.50 (0.19-1.30)  p = 0.1522 |
| Differentiation (Poor) | 1.16 (0.47-2.87)  p = 0.7428 | 0.89 (0.24-3.31)  p = 0.8572 | - | - | - | - |
| Post-operative adjuvant chemotherapy | 1.17 (0.43-3.17)  p = 0.7648 | 0.73 (0.15-3.44)  p = 0.6885 | - | - | - | - |
| TIL (high) | 0.56 (0.35-0.88)  p = 0.0126 | 0.33 (0.17-0.62)  p = 0.0006 | 0.70 (0.12-4.06)  p = 0.6925 | 0.47 (0.10-2.27)  p = 0.3459 | 0.54 (0.36-0.80)  p = 0.0023 | 0.33 (0.20-0.55)  p = 0.0000 |
| TAM (high) | 1.45 (0.85-2.46)  p = 0.1727 | 0.89 (0.45-1.73)  p = 0.7219 | 12.41 (1.94-79.34)  p = 0.0078 | 1.15 (0.32-4.14)  p = 0.8340 | 1.48 (0.94-2.32)  p = 0.0910 | 0.98 (0.59-1.63)  p = 0.9373 |
| MI (high) | 3.40 (1.80-6.41)  p = 0.0002 | 3.93 (1.69-9.14)  p = 0.0015 | 0.89 (0.17-4.59)  p = 0.8881 | 2.92 (0.63-13.46)  p = 0.1700 | 3.12 (1.84-5.30)  p = 0.0000 | 3.36 (1.74-6.48)  p = 0.0003 |
| * From the baseline MRI performed before the start of nCRT | | | | |  |  |

**Table S9. Univariable Cox regression results in the combined cohort.**

| Variable | DFS (HR [95%CI]) | OS (HR [95%CI]) | DFS (C-index [95% CI]) | OS (C-index [95% CI]) |
| --- | --- | --- | --- | --- |
| TIL | 0.54 (0.37-0.79)  p = 0.0013 | 0.35 (0.22-0.56)  p < 0.0001 | 0.59 (0.54-0.64) | 0.64 (0.60-0.68) |
| TAM | 1.85 (1.19-2.87)  p = 0.0061 | 1.36 (0.83-2.23)  p = 0.2171 | 0.57 (0.54-0.61) | 0.54 (0.49-0.60) |
| MI | 2.42 (1.38-1.47)  p = 0.0005 | 2.05 (1.31-1.13)  p = 0.0186 | 0.54 (0.51-0.58) | 0.54 (0.49-0.58) |
| * 95% CIs for C-index were obtained from 10-fold cross validation | | | | |

**Table S10. The number of patients in each group stratified by pre-treatment characteristics, DNA mutation and the change in TIL density.**

|  |  | **Missing** | **Overall** | **TIL^− +^** | **TIL^− −^** | **p** | **Test** |
| --- | --- | --- | --- | --- | --- | --- | --- |
|  |  |  | 78 | 47 | 31 |  |  |
| Age | <=60 | 0 | 35 (44.9) | 22 (46.8) | 13 (41.9) | 0.849 | Chi-squared |
|  | >60 |  | 43 (55.1) | 25 (53.2) | 18 (58.1) |  |  |
| Sex | Female | 0 | 19 (24.4) | 13 (27.7) | 6 (19.4) | 0.571 | Chi-squared |
|  | Male |  | 59 (75.6) | 34 (72.3) | 25 (80.6) |  |  |
| Arm | IrCRT | 0 | 36 (46.2) | 27 (57.4) | 9 (29.0) | 0.026 | Chi-squared |
|  | CRT |  | 42 (53.8) | 20 (42.6) | 22 (71.0) |  |  |
| MRI T Stage | 2.0 | 0 | 2 (2.6) | 1 (2.1) | 1 (3.2) | 0.868 | Chi-squared (warning: expected count < 5) |
|  | 3.0 |  | 65 (83.3) | 40 (85.1) | 25 (80.6) |  |  |
|  | 4.0 |  | 11 (14.1) | 6 (12.8) | 5 (16.1) |  |  |
| MRI N Stage | 0.0 | 0 | 13 (16.7) | 6 (12.8) | 7 (22.6) | 0.522 | Chi-squared |
|  | 1.0 |  | 41 (52.6) | 26 (55.3) | 15 (48.4) |  |  |
|  | 2.0 |  | 24 (30.8) | 15 (31.9) | 9 (29.0) |  |  |
| MSI | MSS | 0 | 78 (100.0) | 47 (100.0) | 31 (100.0) | 1.000 | Chi-squared |
| Meth - Global Mean |  | 47 | 0.4 [0.4,0.5] | 0.4 [0.4,0.5] | 0.4 [0.4,0.5] | 0.901 | Kruskal-Wallis |
| Hypermutation | No | 0 | 77 (98.7) | 46 (97.9) | 31 (100.0) | 1.000 | Fisher's exact |
|  | Unclear |  | 1 (1.3) | 1 (2.1) |  |  |  |
| *POLE_EDM* | 0 | 0 | 77 (98.7) | 46 (97.9) | 31 (100.0) | 1.000 | Fisher's exact |
|  | 1 |  | 1 (1.3) | 1 (2.1) |  |  |  |
| *BRAF_V600E* | 0 | 0 | 77 (98.7) | 47 (100.0) | 30 (96.8) | 0.397 | Fisher's exact |
|  | 1 |  | 1 (1.3) |  | 1 (3.2) |  |  |
| *BRAF_other* | 0 | 0 | 75 (96.2) | 46 (97.9) | 29 (93.5) | 0.560 | Fisher's exact |
|  | 1 |  | 3 (3.8) | 1 (2.1) | 2 (6.5) |  |  |
| *PIK3CA_ex9* | 0 | 0 | 69 (88.5) | 41 (87.2) | 28 (90.3) | 1.000 | Fisher's exact |
|  | 1 |  | 9 (11.5) | 6 (12.8) | 3 (9.7) |  |  |
| *PIK3CA_ex20* | 0 | 0 | 77 (98.7) | 46 (97.9) | 31 (100.0) | 1.000 | Fisher's exact |
|  | 1 |  | 1 (1.3) | 1 (2.1) |  |  |  |
| *PIK3CA_other* | 0 | 0 | 76 (97.4) | 47 (100.0) | 29 (93.5) | 0.155 | Fisher's exact |
|  | 1 |  | 2 (2.6) |  | 2 (6.5) |  |  |
| *NRAS_c1213* | 0 | 0 | 78 (100.0) | 47 (100.0) | 31 (100.0) | 1.000 | Chi-squared |
| *NRAS_c61* | 0 | 0 | 77 (98.7) | 46 (97.9) | 31 (100.0) | 1.000 | Fisher's exact |
|  | 1 |  | 1 (1.3) | 1 (2.1) |  |  |  |
| *NRAS_other* | 0 | 0 | 78 (100.0) | 47 (100.0) | 31 (100.0) | 1.000 | Chi-squared |
| *ACVR1B* | 0 | 0 | 77 (98.7) | 47 (100.0) | 30 (96.8) | 0.397 | Fisher's exact |
|  | 1 |  | 1 (1.3) |  | 1 (3.2) |  |  |
| *ACVR2A* | 0 | 0 | 78 (100.0) | 47 (100.0) | 31 (100.0) | 1.000 | Chi-squared |
| *APC* | 0 | 0 | 17 (21.8) | 11 (23.4) | 6 (19.4) | 0.886 | Chi-squared |
|  | 1 |  | 61 (78.2) | 36 (76.6) | 25 (80.6) |  |  |
| *ARID1A* | 0 | 0 | 73 (93.6) | 45 (95.7) | 28 (90.3) | 0.381 | Fisher's exact |
|  | 1 |  | 5 (6.4) | 2 (4.3) | 3 (9.7) |  |  |
| *ATM* | 0 | 0 | 77 (98.7) | 47 (100.0) | 30 (96.8) | 0.397 | Fisher's exact |
|  | 1 |  | 1 (1.3) |  | 1 (3.2) |  |  |
| *ATR* | 0 | 0 | 78 (100.0) | 47 (100.0) | 31 (100.0) | 1.000 | Chi-squared |
| *BCL9L* | 0 | 0 | 78 (100.0) | 47 (100.0) | 31 (100.0) | 1.000 | Chi-squared |
| *BRAF* | 0 | 0 | 74 (94.9) | 46 (97.9) | 28 (90.3) | 0.295 | Fisher's exact |
|  | 1 |  | 4 (5.1) | 1 (2.1) | 3 (9.7) |  |  |
| *CREBBP* | 0 | 0 | 77 (98.7) | 46 (97.9) | 31 (100.0) | 1.000 | Fisher's exact |
|  | 1 |  | 1 (1.3) | 1 (2.1) |  |  |  |
| *ERBB2* | 0 | 0 | 77 (98.7) | 47 (100.0) | 30 (96.8) | 0.397 | Fisher's exact |
|  | 1 |  | 1 (1.3) |  | 1 (3.2) |  |  |
| *ERBB3* | 0 | 0 | 77 (98.7) | 47 (100.0) | 30 (96.8) | 0.397 | Fisher's exact |
|  | 1 |  | 1 (1.3) |  | 1 (3.2) |  |  |
| *FBXW7* | 0 | 0 | 67 (85.9) | 39 (83.0) | 28 (90.3) | 0.511 | Fisher's exact |
|  | 1 |  | 11 (14.1) | 8 (17.0) | 3 (9.7) |  |  |
| *KRAS* | 0 | 0 | 35 (44.9) | 19 (40.4) | 16 (51.6) | 0.460 | Chi-squared |
|  | 1 |  | 43 (55.1) | 28 (59.6) | 15 (48.4) |  |  |
| *MLH1* | 0 | 0 | 77 (98.7) | 47 (100.0) | 30 (96.8) | 0.397 | Fisher's exact |
|  | 1 |  | 1 (1.3) |  | 1 (3.2) |  |  |
| *MSH3* | 0 | 0 | 76 (97.4) | 45 (95.7) | 31 (100.0) | 0.515 | Fisher's exact |
|  | 1 |  | 2 (2.6) | 2 (4.3) |  |  |  |
| *MSH6* | 0 | 0 | 76 (97.4) | 46 (97.9) | 30 (96.8) | 1.000 | Fisher's exact |
|  | 1 |  | 2 (2.6) | 1 (2.1) | 1 (3.2) |  |  |
| *NF1* | 0 | 0 | 77 (98.7) | 46 (97.9) | 31 (100.0) | 1.000 | Fisher's exact |
|  | 1 |  | 1 (1.3) | 1 (2.1) |  |  |  |
| *NRAS* | 0 | 0 | 77 (98.7) | 46 (97.9) | 31 (100.0) | 1.000 | Fisher's exact |
|  | 1 |  | 1 (1.3) | 1 (2.1) |  |  |  |
| *PCBP1* | 0 | 0 | 76 (97.4) | 46 (97.9) | 30 (96.8) | 1.000 | Fisher's exact |
|  | 1 |  | 2 (2.6) | 1 (2.1) | 1 (3.2) |  |  |
| *PIK3CA* | 0 | 0 | 66 (84.6) | 40 (85.1) | 26 (83.9) | 1.000 | Fisher's exact |
|  | 1 |  | 12 (15.4) | 7 (14.9) | 5 (16.1) |  |  |
| *PIK3R1* | 0 | 0 | 76 (97.4) | 45 (95.7) | 31 (100.0) | 0.515 | Fisher's exact |
|  | 1 |  | 2 (2.6) | 2 (4.3) |  |  |  |
| *PTEN* | 0 | 0 | 78 (100.0) | 47 (100.0) | 31 (100.0) | 1.000 | Chi-squared |
| *RNF43* | 0 | 0 | 78 (100.0) | 47 (100.0) | 31 (100.0) | 1.000 | Chi-squared |
| *SMAD2* | 0 | 0 | 72 (92.3) | 44 (93.6) | 28 (90.3) | 0.677 | Fisher's exact |
|  | 1 |  | 6 (7.7) | 3 (6.4) | 3 (9.7) |  |  |
| *SMAD4* | 0 | 0 | 71 (91.0) | 42 (89.4) | 29 (93.5) | 0.697 | Fisher's exact |
|  | 1 |  | 7 (9.0) | 5 (10.6) | 2 (6.5) |  |  |
| *SOX9* | 0 | 0 | 70 (89.7) | 41 (87.2) | 29 (93.5) | 0.467 | Fisher's exact |
|  | 1 |  | 8 (10.3) | 6 (12.8) | 2 (6.5) |  |  |
| *TCF7L2* | 0 | 0 | 74 (94.9) | 44 (93.6) | 30 (96.8) | 1.000 | Fisher's exact |
|  | 1 |  | 4 (5.1) | 3 (6.4) | 1 (3.2) |  |  |
| *TP53* | 0 | 0 | 22 (28.2) | 14 (29.8) | 8 (25.8) | 0.900 | Chi-squared |
|  | 1 |  | 56 (71.8) | 33 (70.2) | 23 (74.2) |  |  |
| *AMER1* | 0 | 0 | 73 (93.6) | 43 (91.5) | 30 (96.8) | 0.643 | Fisher's exact |
|  | 1 |  | 5 (6.4) | 4 (8.5) | 1 (3.2) |  |  |
| *AXIN2* | 0 | 0 | 78 (100.0) | 47 (100.0) | 31 (100.0) | 1.000 | Chi-squared |
| *B2M* | 0 | 0 | 75 (96.2) | 44 (93.6) | 31 (100.0) | 0.272 | Fisher's exact |
|  | 1 |  | 3 (3.8) | 3 (6.4) |  |  |  |
| *BMPR2* | 0 | 0 | 77 (98.7) | 46 (97.9) | 31 (100.0) | 1.000 | Fisher's exact |
|  | 1 |  | 1 (1.3) | 1 (2.1) |  |  |  |
| *BUB1B* | 0 | 0 | 78 (100.0) | 47 (100.0) | 31 (100.0) | 1.000 | Chi-squared |
| *CASP8* | 0 | 0 | 78 (100.0) | 47 (100.0) | 31 (100.0) | 1.000 | Chi-squared |
| *CD58* | 0 | 0 | 77 (98.7) | 47 (100.0) | 30 (96.8) | 0.397 | Fisher's exact |
|  | 1 |  | 1 (1.3) |  | 1 (3.2) |  |  |
| *CDC27* | 0 | 0 | 78 (100.0) | 47 (100.0) | 31 (100.0) | 1.000 | Chi-squared |
| *CDK8* | 0 | 0 | 78 (100.0) | 47 (100.0) | 31 (100.0) | 1.000 | Chi-squared |
| *CDKN2A* | 0 | 0 | 78 (100.0) | 47 (100.0) | 31 (100.0) | 1.000 | Chi-squared |
| *CTNNB1* | 0 | 0 | 76 (97.4) | 46 (97.9) | 30 (96.8) | 1.000 | Fisher's exact |
|  | 1 |  | 2 (2.6) | 1 (2.1) | 1 (3.2) |  |  |
| *ELF3* | 0 | 0 | 77 (98.7) | 46 (97.9) | 31 (100.0) | 1.000 | Fisher's exact |
|  | 1 |  | 1 (1.3) | 1 (2.1) |  |  |  |
| *EP300* | 0 | 0 | 77 (98.7) | 46 (97.9) | 31 (100.0) | 1.000 | Fisher's exact |
|  | 1 |  | 1 (1.3) | 1 (2.1) |  |  |  |
| *FGFR3* | 0 | 0 | 78 (100.0) | 47 (100.0) | 31 (100.0) | 1.000 | Chi-squared |
| *FLT3* | 0 | 0 | 78 (100.0) | 47 (100.0) | 31 (100.0) | 1.000 | Chi-squared |
| *GNAS* | 0 | 0 | 77 (98.7) | 46 (97.9) | 31 (100.0) | 1.000 | Fisher's exact |
|  | 1 |  | 1 (1.3) | 1 (2.1) |  |  |  |
| *HDLBP* | 0 | 0 | 78 (100.0) | 47 (100.0) | 31 (100.0) | 1.000 | Chi-squared |
| *HLA.A* | 0 | 0 | 77 (98.7) | 46 (97.9) | 31 (100.0) | 1.000 | Fisher's exact |
|  | 1 |  | 1 (1.3) | 1 (2.1) |  |  |  |
| *HLA.B* | 0 | 0 | 78 (100.0) | 47 (100.0) | 31 (100.0) | 1.000 | Chi-squared |
| *HRAS* | 0 | 0 | 77 (98.7) | 46 (97.9) | 31 (100.0) | 1.000 | Fisher's exact |
|  | 1 |  | 1 (1.3) | 1 (2.1) |  |  |  |
| *IDH1* | 0 | 0 | 78 (100.0) | 47 (100.0) | 31 (100.0) | 1.000 | Chi-squared |
| *IGF2* | 0 | 0 | 78 (100.0) | 47 (100.0) | 31 (100.0) | 1.000 | Chi-squared |
| *IRS2* | 0 | 0 | 78 (100.0) | 47 (100.0) | 31 (100.0) | 1.000 | Chi-squared |
| *MAP2K4* | 0 | 0 | 77 (98.7) | 46 (97.9) | 31 (100.0) | 1.000 | Fisher's exact |
|  | 1 |  | 1 (1.3) | 1 (2.1) |  |  |  |
| *MBD6* | 0 | 0 | 78 (100.0) | 47 (100.0) | 31 (100.0) | 1.000 | Chi-squared |
| *MET* | 0 | 0 | 78 (100.0) | 47 (100.0) | 31 (100.0) | 1.000 | Chi-squared |
| *MSH2* | 0 | 0 | 78 (100.0) | 47 (100.0) | 31 (100.0) | 1.000 | Chi-squared |
| *MYC* | 0 | 0 | 78 (100.0) | 47 (100.0) | 31 (100.0) | 1.000 | Chi-squared |
| *PMS2* | 0 | 0 | 78 (100.0) | 47 (100.0) | 31 (100.0) | 1.000 | Chi-squared |
| *POLE* | 0 | 0 | 78 (100.0) | 47 (100.0) | 31 (100.0) | 1.000 | Chi-squared |
| *PPP2R1A* | 0 | 0 | 78 (100.0) | 47 (100.0) | 31 (100.0) | 1.000 | Chi-squared |
| *RAF1* | 0 | 0 | 77 (98.7) | 46 (97.9) | 31 (100.0) | 1.000 | Fisher's exact |
|  | 1 |  | 1 (1.3) | 1 (2.1) |  |  |  |
| *RBM10* | 0 | 0 | 77 (98.7) | 47 (100.0) | 30 (96.8) | 0.397 | Fisher's exact |
|  | 1 |  | 1 (1.3) |  | 1 (3.2) |  |  |
| *SMAD3* | 0 | 0 | 77 (98.7) | 46 (97.9) | 31 (100.0) | 1.000 | Fisher's exact |
|  | 1 |  | 1 (1.3) | 1 (2.1) |  |  |  |
| *SMARCA4* | 0 | 0 | 74 (94.9) | 44 (93.6) | 30 (96.8) | 1.000 | Fisher's exact |
|  | 1 |  | 4 (5.1) | 3 (6.4) | 1 (3.2) |  |  |
| *TGIF1* | 0 | 0 | 77 (98.7) | 47 (100.0) | 30 (96.8) | 0.397 | Fisher's exact |
|  | 1 |  | 1 (1.3) |  | 1 (3.2) |  |  |
| *WBP1* | 0 | 0 | 78 (100.0) | 47 (100.0) | 31 (100.0) | 1.000 | Chi-squared |
| *ZFP36L2* | 0 | 0 | 74 (94.9) | 45 (95.7) | 29 (93.5) | 1.000 | Fisher's exact |
|  | 1 |  | 4 (5.1) | 2 (4.3) | 2 (6.5) |  |  |

#
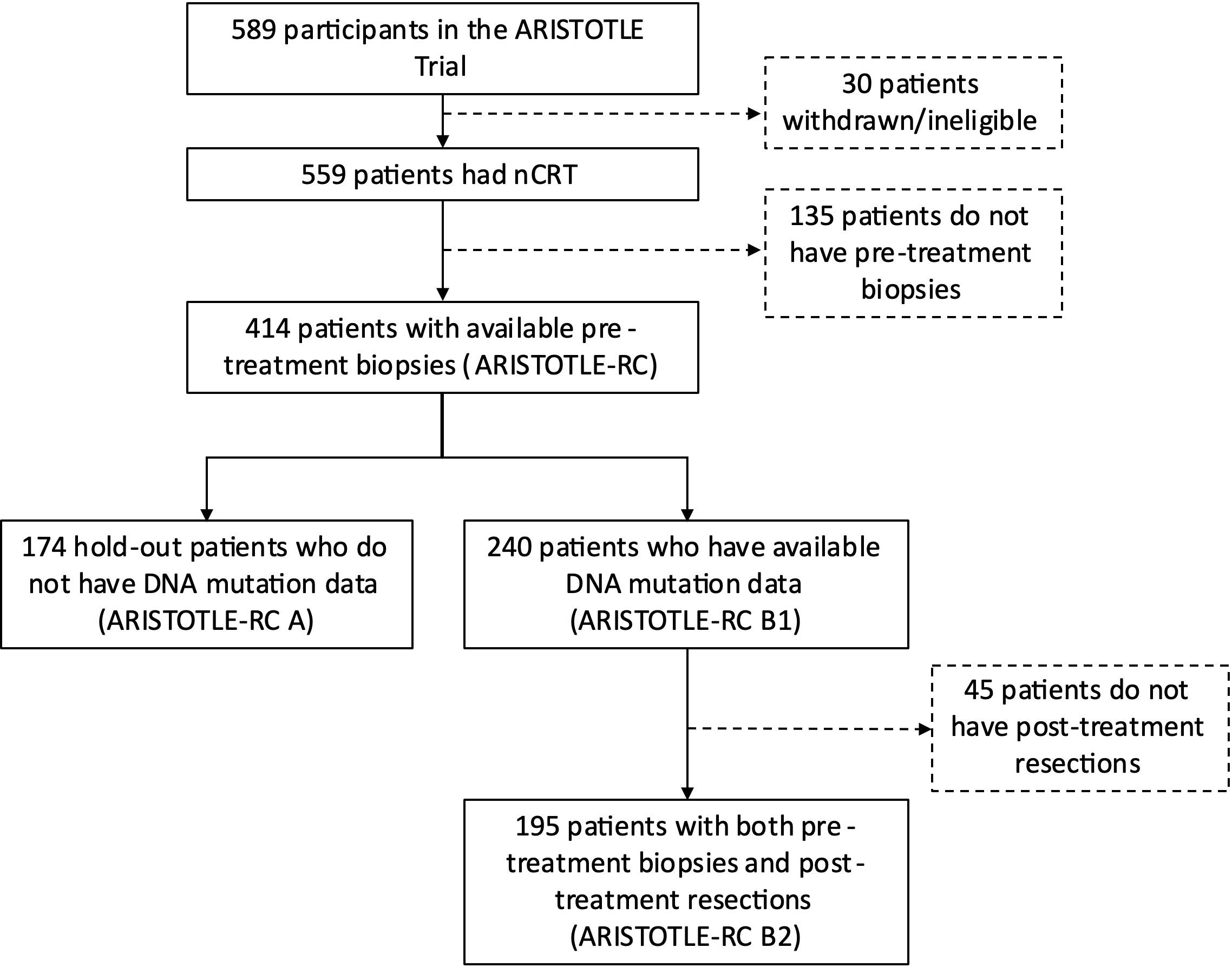
**Supplementary Figures**

**Figure S1. Cohort selection diagram of the ARISTOTLE trial participants.**


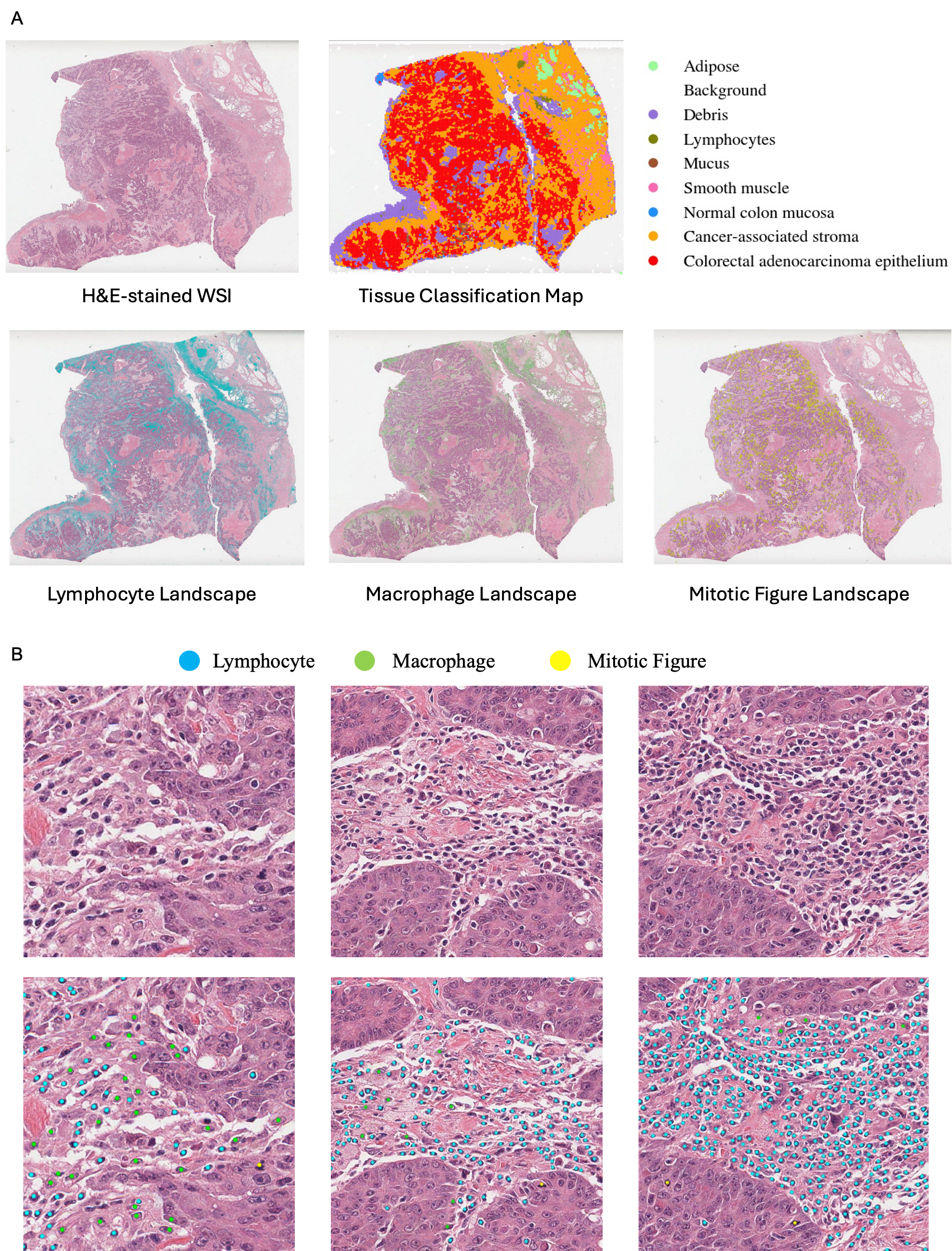


**Figure S2. Illustration of the outputs of the AI framework.** A. Examples of the tissue classification map and cell landscapes predicted by the AI models. B. Examples of lymphocytes (blue), macrophages (green) and mitotic figures (yellow) detected by the AI.

**
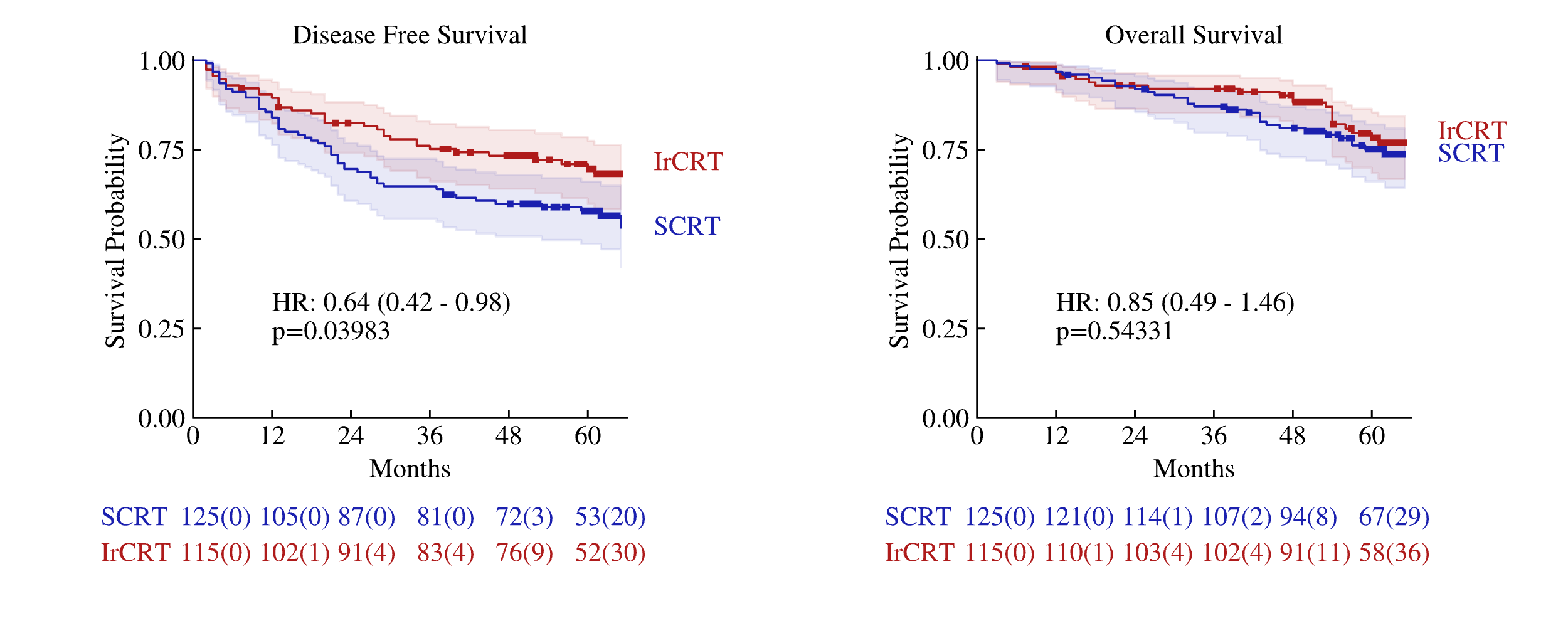
**

**Figure S3. The Kaplan-Meier curves of DFS (left) and OS (right) of patients in IrCRT and SCRT arm.**

**
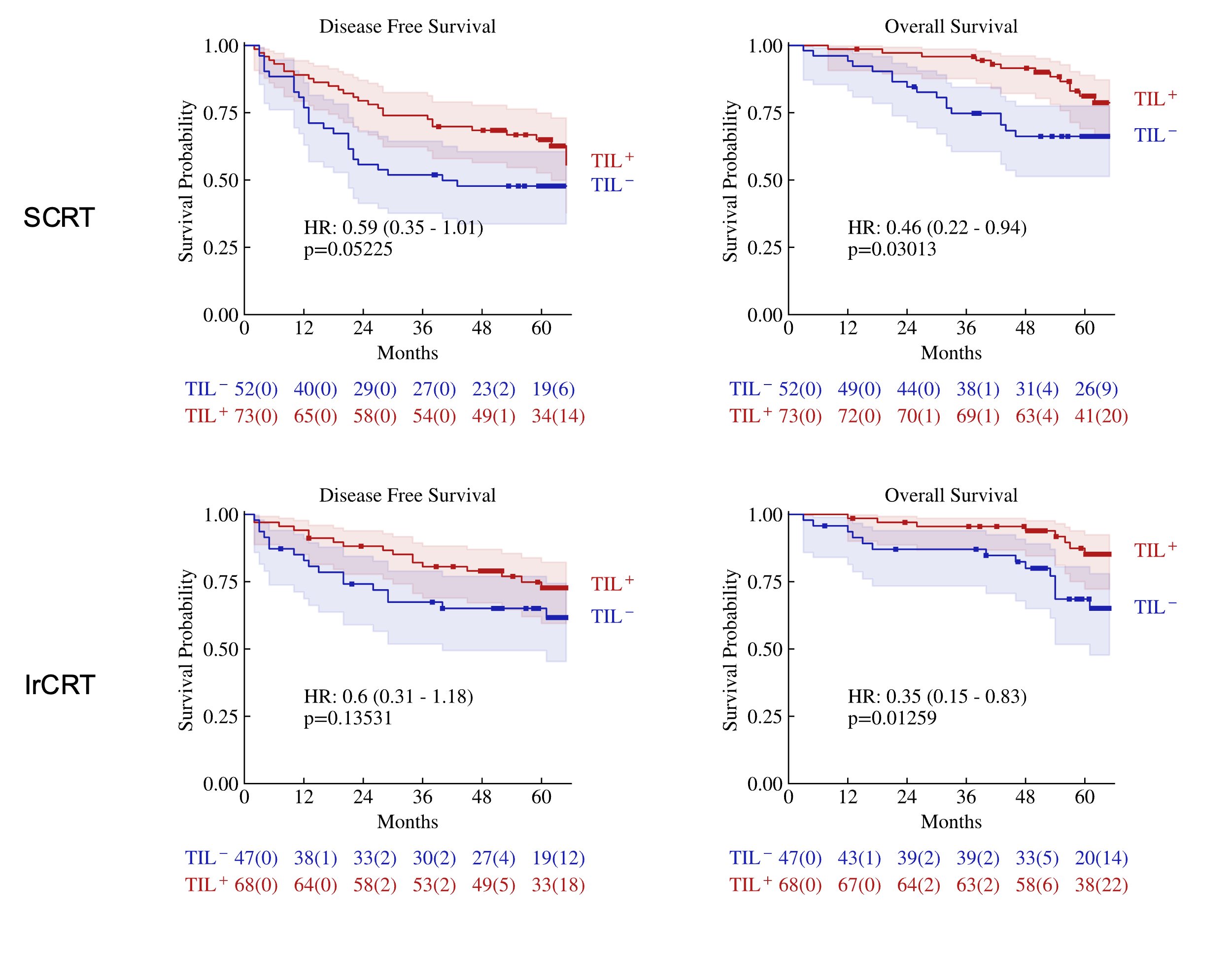
**

**Figure S4. The Kaplan-Meier curves of DFS (left) and OS (right) of TIL^+^ and TIL^–^ patients in the SCRT arm and IrCRT arm.**

**
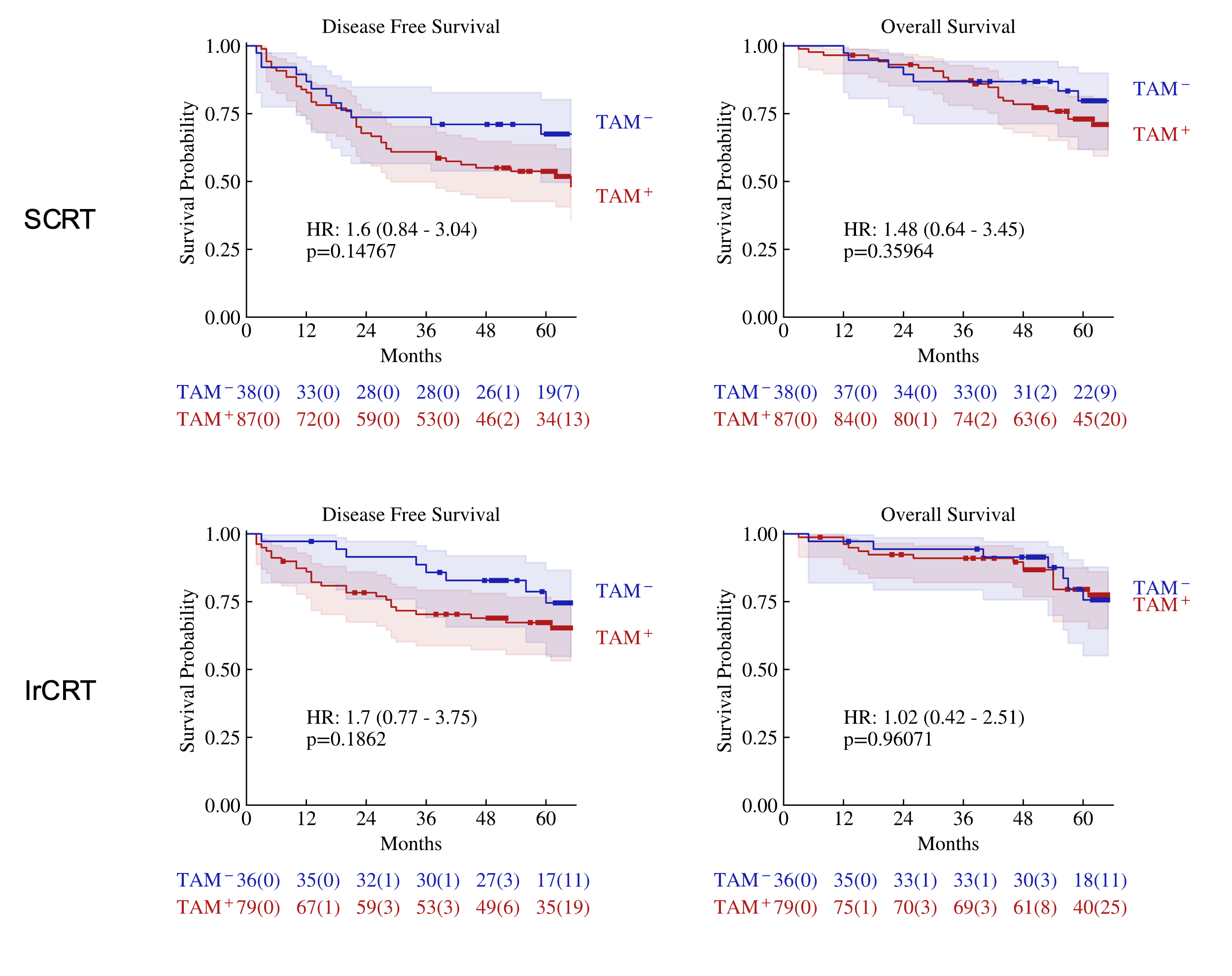
**

**Figure S5. The Kaplan-Meire curves of DFS (left) and OS (right) of TAM^+^ and TAM^–^ patients in the SCRT arm and IrCRT arm.**

**
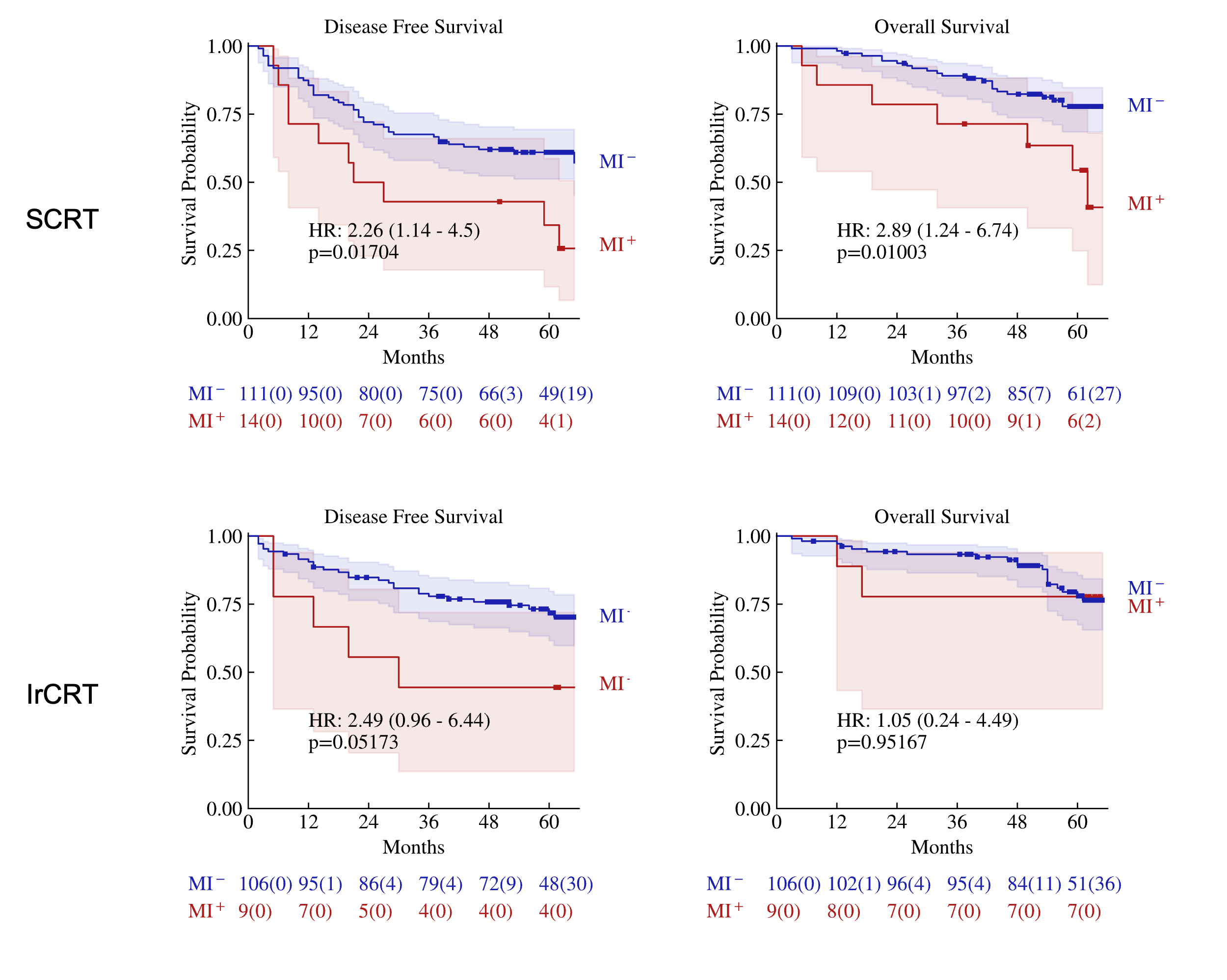
**

**Figure S6. The Kaplan-Meier curves of DFS (left) and OS (right) of MI^+^ and MI^–^ patients in the SCRT arm and IrCRT arm.**

**
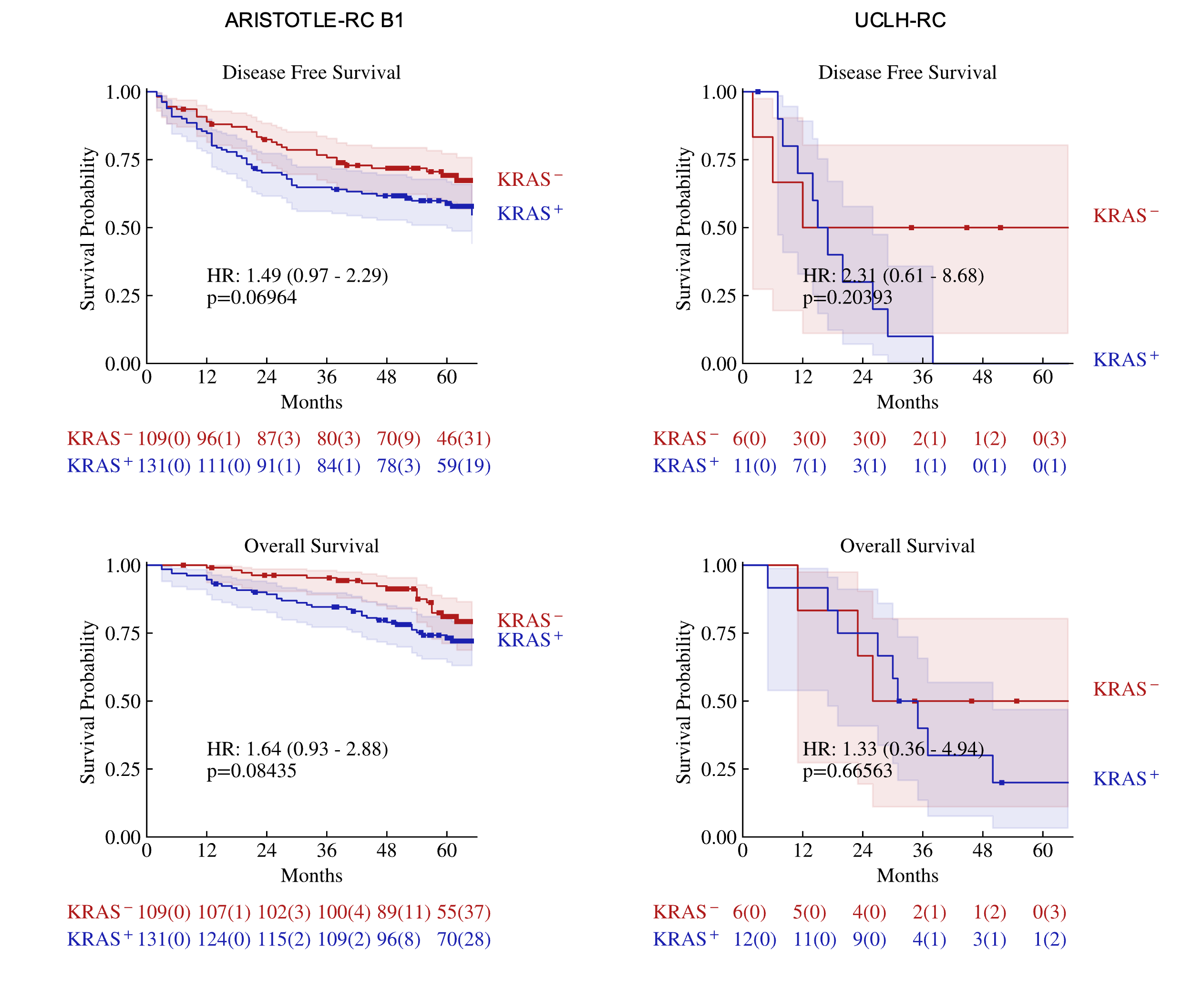
**

**Figure S7. The Kaplan-Meier curves of DFS and OS of KRAS^+^ and KRAS^–^ patients in ARISTOTLE_RC (left) and UCLH-RC (right).**


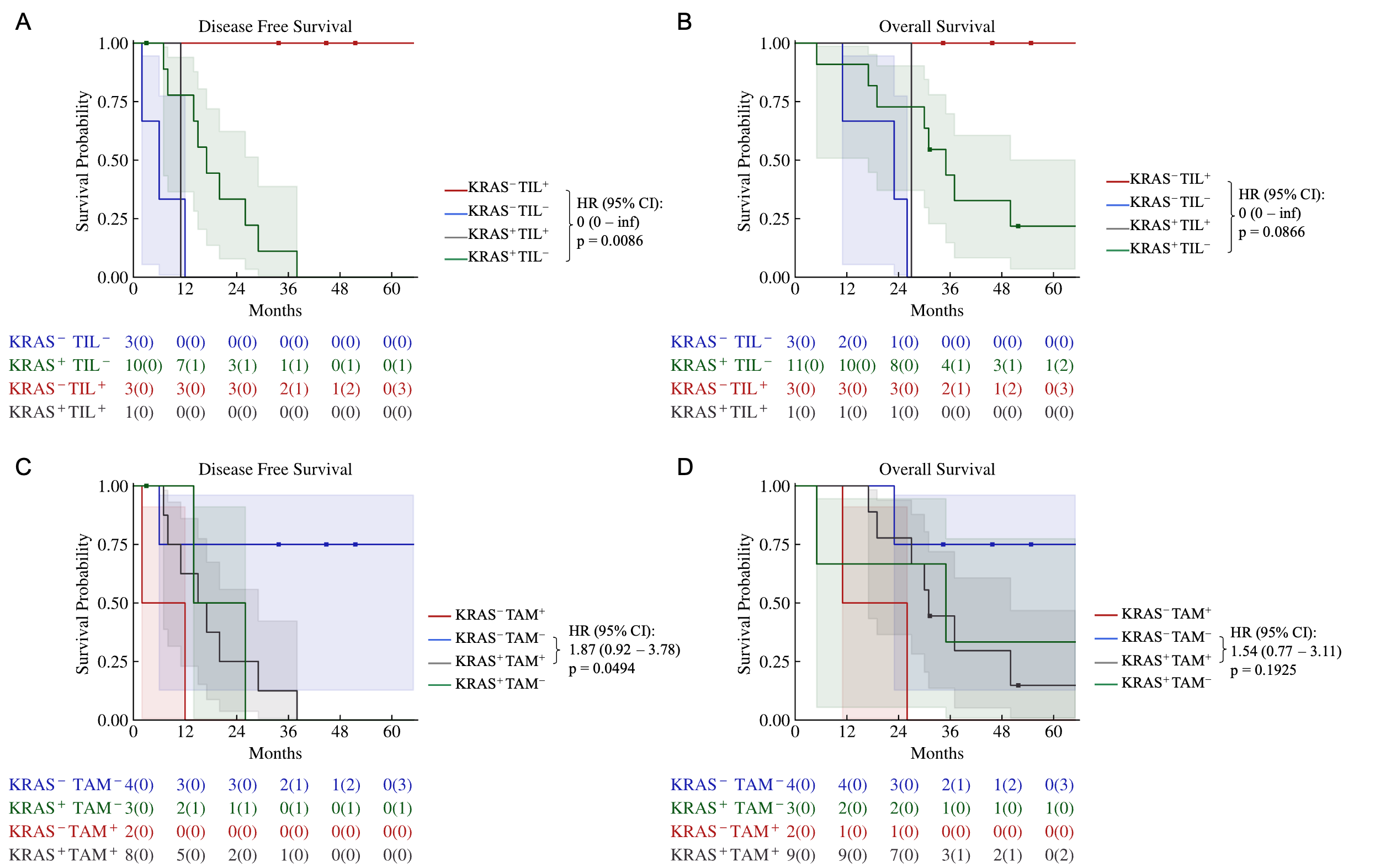


**Figure S8. The Kaplan-Meier curves of DFS and OS of the four subgroups stratified by KRAS mutation and TIL/TAM density in the 18 patients with available KRAS data in UCLH-RC.**


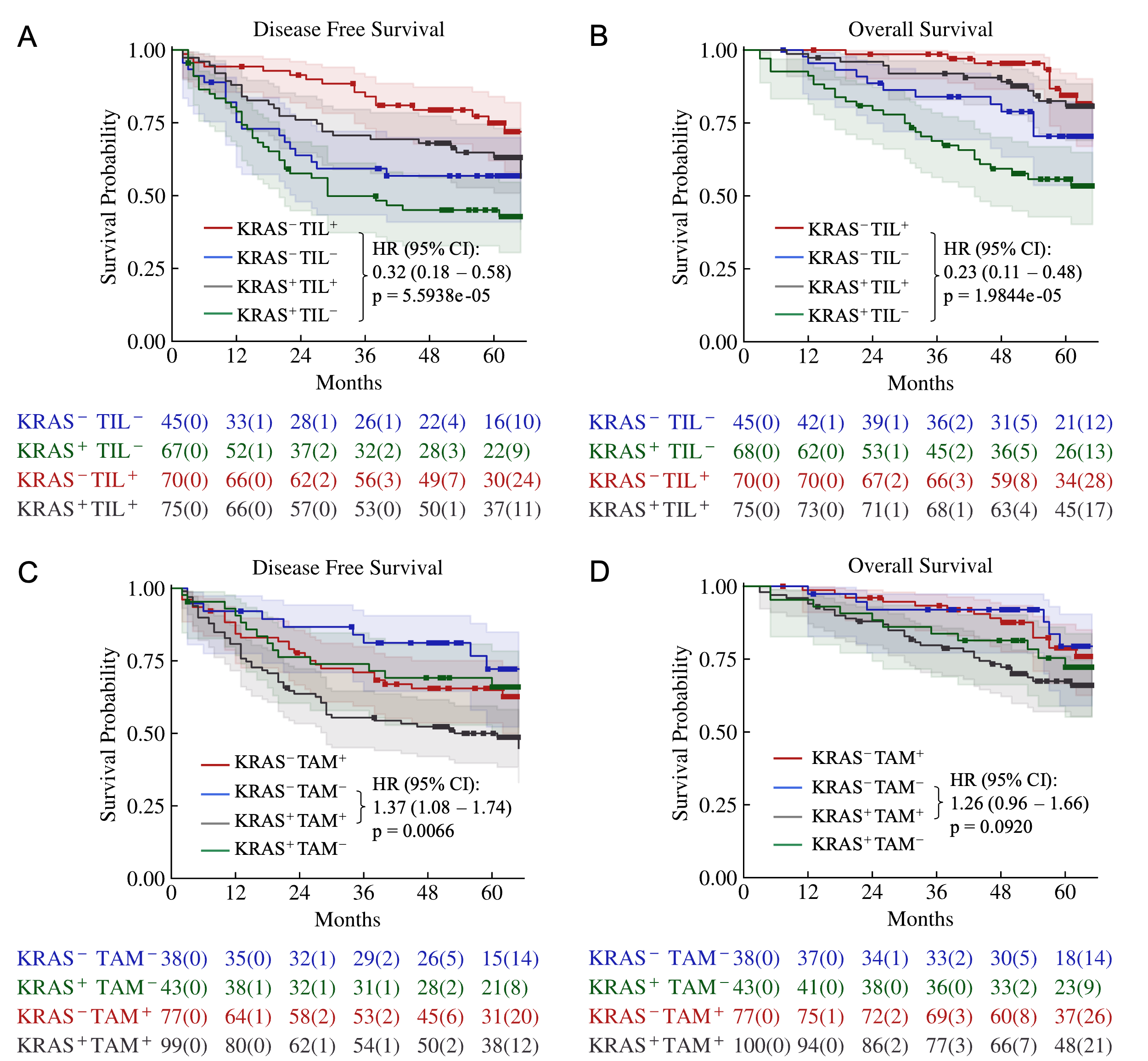


**Figure S9. The Kaplan-Meier curves of DFS and OS of the four subgroups stratified by KRAS mutation and TIL/TAM density in the combined cohort (240 patients in ARISTOTLE-RC and 18 patients in UCLH-RC).**


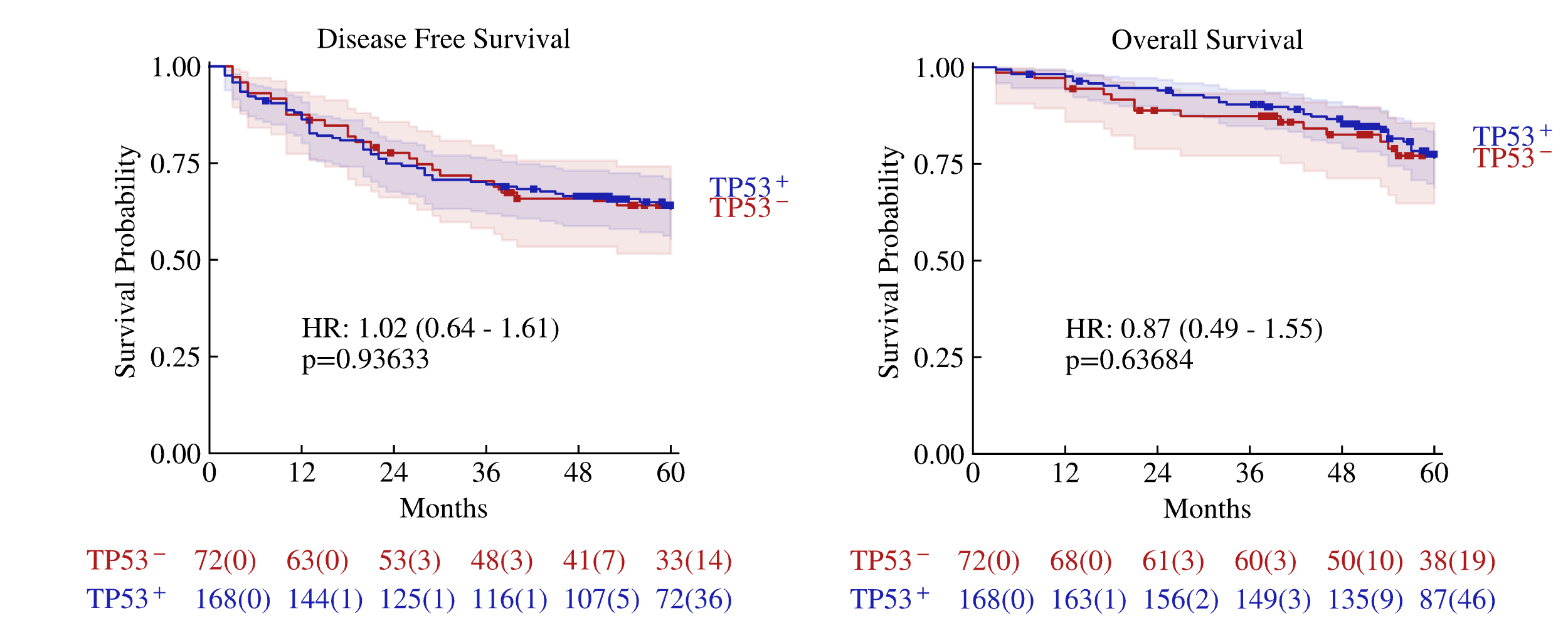


**Figure S10. The Kaplan-Meier curves of DFS (left) and OS (right) of TP53^+^ and TP53^–^ patients in ARISTOTLE_RC.**

#
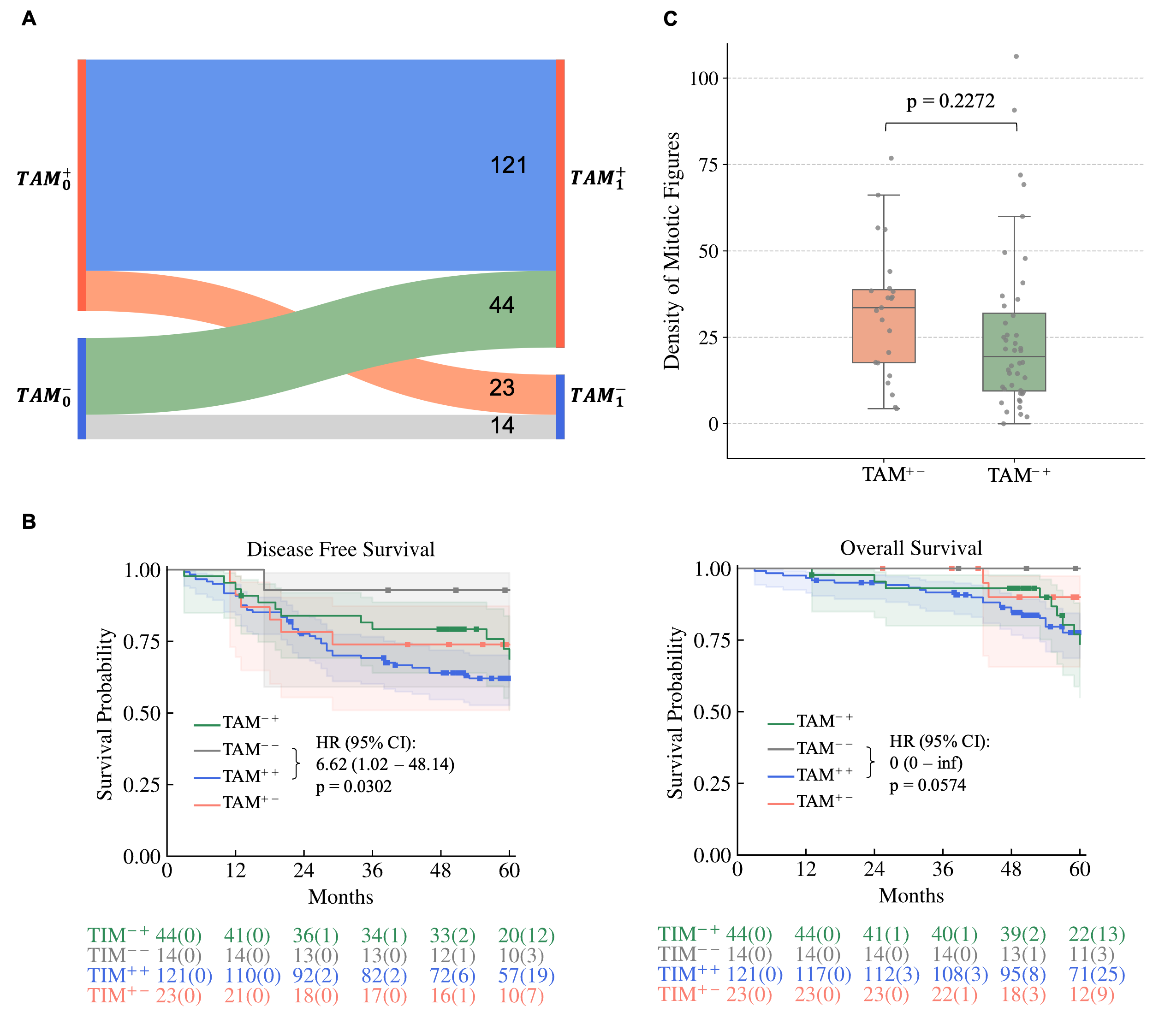


**Figure S11. The patient re-stratification after the nCRT.** A. The four re-stratification routes and the numbers in each group; B. The Kaplan-Meire curves of DFS (left) and OS (right) of TAM^+ +^, TAM^+ –^, TAM^– +^ and TAM ^–^. C. The density of mitotic figures in the TAM^+ –^ and TAM^– +^ patients.
